# Progressive cardiac phenotypes and reduced reversibility from long-term CUG_exp_ RNA expression in a DM1 mouse model

**DOI:** 10.1172/jci.insight.204278

**Published:** 2026-03-19

**Authors:** Rong-Chi Hu, Mohammadreza Tabary, Xander H.T. Wehrens, Thomas A. Cooper

**Affiliations:** 1 Department of Pathology & Immunology,; 2 Department of Medicine (Cardiology Section),; 3 Department of Integrative Physiology, and; 4 Department of Molecular & Cellular Biology, Baylor College of Medicine, Houston, Texas, USA.

**Keywords:** Cardiology, Genetics, Cardiovascular disease, Genetic diseases, Molecular biology

## Abstract

Myotonic dystrophy type 1 (DM1) is caused by an expanded CTG repeat in the *DMPK* gene, resulting in mutant transcripts that form expanded CUG (CUG_exp_) RNA foci and sequester muscleblind-like (MBNL) RNA-binding proteins. DM1 is multisystemic, with progressive worsening of disease manifestations in affected tissues. Disease progression is attributed to somatic expansion of the CTG repeats with age, resulting in production of CUG_exp_ RNA with enhanced intrinsic toxicity due to increased MBNL sequestration. To determine the degree to which cardiac disease progression can occur independently of repeat expansion, we used a transgenic DM1 mouse model with inducible heart-specific expression of a stable, interrupted 960-CUG-repeat RNA. Sustained CUG_exp_ RNA expression caused progressive cardiac enlargement, contractile dysfunction, conduction delay, myocardial fibrosis, and reduced survival, while MBNL-dependent splicing defects remained static, consistent with the stable repeat length. We also determined the degree of reversibility after different periods of CUG_exp_ RNA expression by shutting off the repeat-containing transgene. Suppression of CUG_exp_ RNA expression rescued cardiac abnormalities, but reversibility declined with longer exposure to the toxic RNA. These findings demonstrate that prolonged expression of stable CUG_exp_ RNA drives progressive cardiac pathology, revealing a mechanism of disease progression in DM1 in addition to somatic expansion.

## Abstract 2

This manuscript describes a mouse model for myotonic dystrophy type 1 that recapitulates cardiac disease progression, demonstrates reversibility, and highlights the benefits of early intervention

## Introduction

Myotonic dystrophy type 1 (DM1) is an autosomal dominant disease caused by an unstable expanded CTG repeat tract in the 3′ untranslated region of the *DMPK* gene, with pathogenic alleles ranging from 50 to over 4000 CTG repeats (1–3). DM1 is the most common cause of adult-onset muscular dystrophy and disease manifestations are multisystemic, affecting skeletal muscle, heart, central nervous system, and gastrointestinal tissues (4). Pathogenesis results from the expanded CUG repeat (CUG_exp_) RNA expressed from the mutant allele that sequesters members of the muscleblind-like (MBNL) family of RNA-binding proteins (RBPs), leading to their loss of function. MBNL proteins regulate a network of pre-mRNA processing changes during postnatal development, the best characterized being alternative splicing transitions from fetal to adult patterns. The loss of MBNL activities results in misexpression of fetal protein isoforms unable to fulfill the functions required in adult tissues, causing DM1 features (5–9).

Cardiac manifestations impact the majority of DM1-affected individuals, presenting predominantly as conduction abnormalities and life-threatening arrhythmias, accounting for 25% mortality and the second leading cause of death in DM1 (10–12). The prevalence and severity of DM1 cardiac features correlate with age, CTG repeat length, and sex, being more severe in male individuals (12–17). Cardiac conduction abnormalities, seen in up to 75% of adult DM1 cases, include electrocardiographic (ECG) abnormalities, commonly prolongation of the PR, QRS, and QTc intervals, atrioventricular (AV) blocks, and bundle branch block (BBB) (12, 18–21). Sinus-node dysfunction and atrial fibrillation, flutter, and tachycardia are the most common arrhythmias in DM1, while ventricular arrhythmias are less prevalent but potentially life-threatening (10, 12, 18, 19, 22). Late-stage disease is characterized by fibrosis, myocyte hypertrophy, focal fatty infiltration, and lymphocytic infiltration that occur along the conduction system contributing to arrhythmias (21, 23). Affected individuals can also exhibit functional and structural abnormalities, including reduced left ventricular (LV) systolic function, LV hypertrophy, LV dilatation, and reduced right ventricular function (10, 24); however, the delayed conduction and associated arrhythmias, especially AV blocks and ventricular tachyarrhythmias, are the main cause of cardiac-associated morbidity and mortality (10, 12, 18, 19, 22).

The length of the expanded CTG repeat correlates directly with disease severity and inversely with age of onset (25). Genetic anticipation occurs in DM1, in which the disease severity increases, and the age of onset decreases in successive generations as a result of the increasing size of the transmitted repeat expansion (26, 27). In addition, DM1 is a progressive disease characterized by age-dependent increases in severity, including skeletal muscle and cardiac conduction manifestations (17, 20, 28). The hypothesized mechanism for disease progression is somatic instability of the CTG repeat, for which increased repeat length results in expression of *DMPK* mRNAs with longer CUG tracts and increased intrinsic toxicity due to progressive MBNL loss of function. This hypothesis is supported by 3%–5% of individuals found to carry variant repeats with interruptions, which reduce somatic instability and correlates with slower disease progression (29–31). However, the degree to which other factors contribute to disease progression, especially within the different tissues affected, is unknown.

In this study, we tested the progression of DM1 cardiac pathophysiological and molecular manifestations during long-term expression of CUG_exp_ RNA with stable CUG repeat number in mouse heart. We also tested the reversibility of cardiac features after halting CUG_exp_ RNA expression at different time points. We used a bitransgenic mouse model (CUG960) containing the TREDT960I and MHCrtTA transgenes for doxycycline-inducible (dox-inducible) and heart-specific expression of a human *DMPK* RNA containing 960 interrupted CUG repeats (32, 33). CUG960 mice, induced to express CUG_exp_ RNA, reproduce clinical and molecular abnormalities consistent with DM1, including conduction delays, propensity for arrhythmias, nuclear RNA foci with MBNL colocalization, and misregulated splicing (32, 33). In addition, these phenotypes are reversed after 2 months of CUG_exp_ RNA expression by halting dox administration to turn off the transgene (32). Importantly, the interrupted 960 CTG repeats in the *DMPK* transgene were demonstrated to be stable based on a Southern blot analysis of genomic DNA that produced a single band of the expected size after multiple generations (data not shown). This transgene allows us to study cardiac disease progression with long-term CUG_exp_ RNA expression without repeat expansion.

Mice with 2, 8, and 14 months of CUG_exp_ RNA expression showed progressive worsening of cardiac phenotypes, including reduced contractile function, prolonged cardiac conduction intervals, and increased frequency of cardiac arrhythmias compared with age-matched control cohorts expressing only rtTA and on dox. In addition, the progression of cardiac morphological changes detected by echocardiography (echo) suggested an initial hypertrophic response that progressed to enlarged ventricular chambers resembling a dilated phenotype. We also observed late-stage cardiac fibrosis and reduced lifespan, with increased prevalence in males. In contrast with increased severity of physiological phenotypes, the severe misregulated splicing observed at 2 months did not progress, which is consistent with stable CTG repeat length and no change in MBNL sequestration and loss of function.

To determine the reversibility of phenotypes induced by CUG_exp_ RNA for different lengths of time, animals were taken off dox for 2 months after 2, 6, and 12 months of CUG_exp_ RNA expression. While alternative splicing changes were completely reversed in all cohorts, animals with 12 months of CUG_exp_ RNA expression did not show reversibility of the structural and conduction defects to the same extent as animals with shorter CUG_exp_ RNA expression times. Together, the results demonstrate progression of cardiac defects with long-term CUG_exp_ RNA expression and reduced reversibility of abnormal cardiac features with long-term expression of stable CTG repeats. These findings highlight the importance of timely treatment in patients with DM1 to avoid irreversible cardiac complications.

## Results

**Figure 1:**
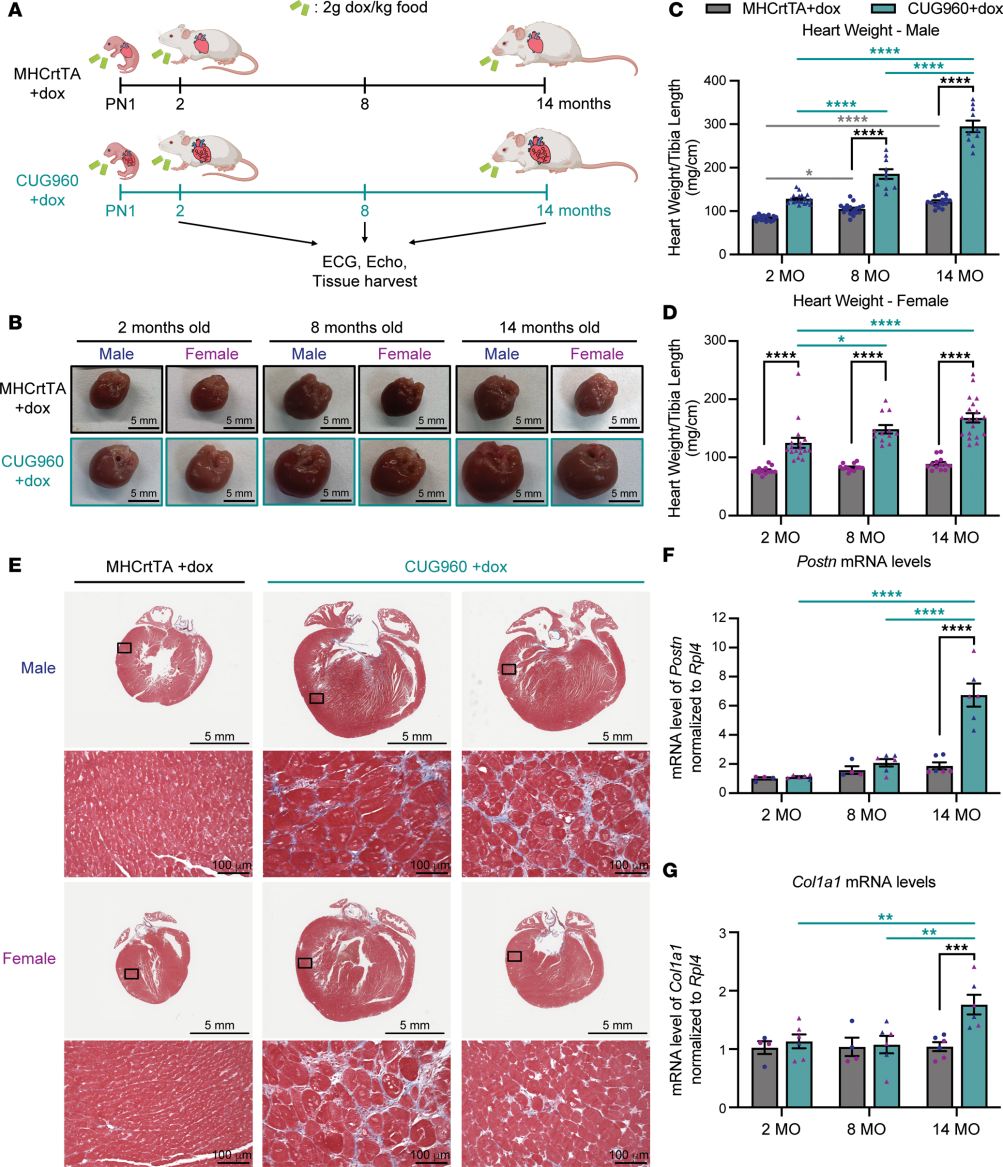
Long-term expression of CUG_exp_ RNA resulted in progressive heart enlargement and cardiac fibrosis. (**A**) Illustration of the experimental timeline and cohorts. (**B**) Representative images of hearts from male and female CUG960 +dox and MHCrtTA +dox mice at 2-, 8-, and 14-month time points. Scale bars: 5 mm. The heart weight/tibia length ratio of (**C**) male and (**D**) female MHCrtTA +dox and CUG960 +dox mice at 2, 8, and 14 months of age (*n* ≥ 10 per time point and sex). (**E**) Representative images of trichrome staining from 4-chamber heart sections of CUG960 +dox and MHCrtTA +dox mice at 14 months of age. Three animals were examined per sex per group. Section thickness: 4 μm. Scale bars: 5 mm (whole-heart images) and 100 μm (zoomed-in images). (**F**) *Postn* and (**G**) *Col1a1* mRNA levels in left ventricular tissues of CUG960 +dox and MHCrtTA +dox mice at 2, 8, and 14 months of age. Males (blue symbols) and females (purple symbols) are indicated. *n* = 6 per time point (3 males and 3 females). Data represent the mean ± SEM and were analyzed using 2-way ANOVA followed by Tukey’s multiple-comparison test. *P < 0.05; **P < 0.01; ***P < 0.001; ****P < 0.0001.

To determine the impact of long-term CUG_exp_ RNA expression in the presence of stable repeat length, we induced the CUG_exp_ RNA in CUG960 mice (CUG960 +dox) on postnatal day 1 (PN1) by feeding 2 g dox/kg chow to nursing dams and kept animals on dox for 2, 8, and 14 months. To control for changes due to normal aging and potential effects caused by rtTA expression and long-term dox administration, hemizygous MHCrtTA mice were given the dox chow (MHCrtTA +dox) following the same timeline as the CUG960 +dox cohort. We performed echo in short-axis view and surface electrocardiography (ECG) on the same animals at 2, 8, and 14 months of age for both CUG960 +dox and MHCrtTA +dox cohorts. Heart tissues were collected for each time point to analyze progression of histological, cellular, and molecular changes in DM1-associated cardiac defects (Figure 1A).

### Long-term CUG_exp_ RNA expression causes cardiac hypertrophy and fibrosis.

As we have shown previously (32, 33), both male and female CUG960 +dox mice develop enlarged hearts and significantly increased heart weight/tibia length (HW/TL) ratios compared with MHCrtTA +dox controls after 2 months of CUG_exp_ RNA expression (Figure 1, B–D, and Supplemental Figure 1, A and B; supplemental material available online with this article; https://doi.org/10.1172/jci.insight.204278DS1). Moreover, the HW/TL ratio of CUG960 +dox mice significantly increased from 2 to 8 months and increased further from 8 to 14 months (Figure 1, C and D), with male mice showing a larger increase than females (Supplemental Figure 1, C and D). We also observed significant increases in HW/TL ratios between 2-month and 8- or 14-month time points in male MHCrtTA +dox control mice due to aging, as expected (34). However, the increase in CUG960 +dox mice was much larger compared with the MHCrtTA +dox control cohort (Supplemental Figure 1, C and D), indicating that the increased HW/TL ratio in CUG960 +dox mice was mainly due to long-term CUG_exp_ RNA expression rather than aging.

Myocardial fibrosis with fat infiltration as well as myocyte hypertrophy and degeneration have been identified in DM1 (23, 35, 36). Trichrome staining was performed on 4-chamber cross sections of hearts from all time points to evaluate the cardiac histopathological features of animals with long-term CUG_exp_ RNA repeat expression in CUG960 +dox compared to MHCrtTA +dox controls. Histological analysis identified cardiac fibrosis and cardiomyocyte hypertrophy in left ventricles of CUG960 +dox mice with CUG_exp_ RNA expression for 14 months (Figure 1E), but not in 14-month MHCrtTA +dox controls or animals expressing CUG_exp_ RNA for 2 months or 8 months (Supplemental Figure 2). Notably, fibrosis was consistently found in males, while less consistent and prominent in females (Figure 1E). Moreover, mRNA levels of *Postn* and *Col1a1*, two fibrosis markers (37, 38), were increased in left ventricular tissues of 14-month-old male and female CUG960 +dox mice compared with MHCrtTA +dox mice, but not in 2- or 8-month-old CUG960 +dox mice (Figure 1, F and G).

Together, the data demonstrate progressive disease with long-term expression of stable CUG_exp_ RNA in CUG960 +dox mice, including increased heart size, cardiac fibrosis, and cardiomyocyte hypertrophy compared with control aging cohorts differing only by the absence of CUG_exp_ RNA.

### Long-term CUG_exp_ RNA expression causes an initial hypertrophic response followed by cardiac dilation and decreased cardiac function.

**Figure 2:**
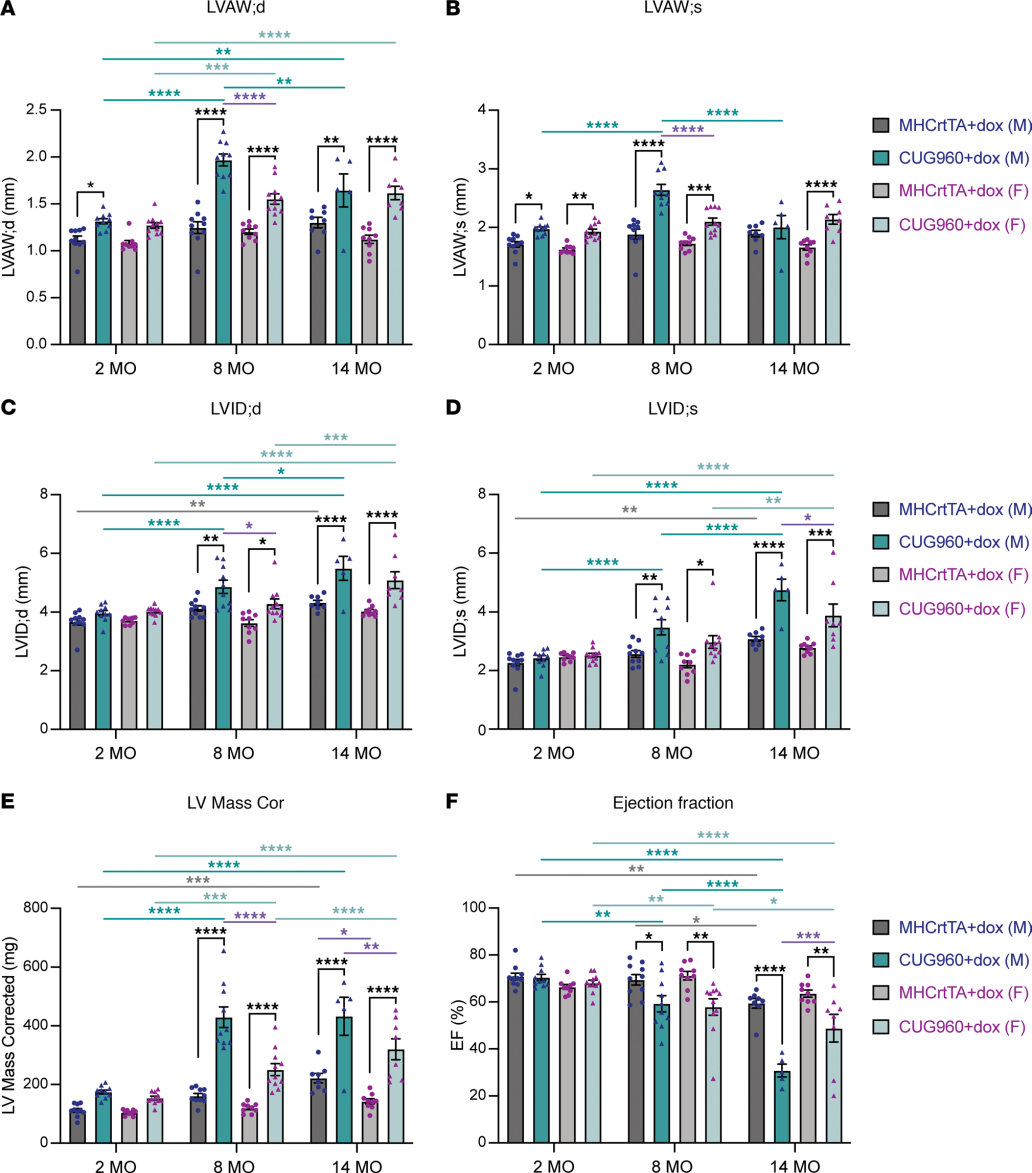
CUG960 +dox mice developed dilated cardiac phenotypes and impaired contractile function with long-term CUG_exp_ RNA expression. M-mode echocardiography was performed on the same CUG960 +dox and MHCrtTA +dox mice at 2, 8, and 14 months of age to evaluate (**A**) left ventricle anterior wall thickness in diastole (LVAW;d), (**B**) left ventricle anterior wall thickness in systole (LVAW;s), (**C**) left ventricle internal diameter in diastole (LVID;d), (**D**) left ventricle internal diameter in systole (LVID;s), (**E**) left ventricle mass, and (**F**) ejection fraction (EF). Males (blue symbols) and females (purple symbols) are indicated. MHCrtTA +dox: 10 males and 9 females. CUG960 +dox: 10 males and 11 females. Data represent the mean ± SEM and were analyzed using 2-way ANOVA followed by Tukey’s multiple-comparison test. *P < 0.05; **P < 0.01; ***P < 0.001; ****P < 0.0001.

M-mode echo showed that cardiac structural and functional parameters in CUG960 +dox mice were significantly altered compared with the MHCrtTA +dox control mice, especially at 8 and 14 months of age (Figure 2 and Supplemental Figure 3). LV wall thickness of male CUG960 +dox mice was significantly increased compared with MHCrtTA +dox controls starting at 2 months of age (Figure 2, A and B, and Supplemental Figure 3, A and B), while LV internal diameter (LVID), volume, and LV mass from male CUG960 +dox mice were significantly increased starting at 8 months (Figure 2, C–E, and Supplemental Figure 3, C and D). Ejection fraction (EF) and fractional shortening (FS) showed no significant decreases between male CUG960 +dox and MHCrtTA +dox until the 8-month time point, temporally correlating with chamber dilation (Figure 2F and Supplemental Figure 3E). Additionally, within the CUG960 +dox cohort, LV mass and LV wall thickness of the male animals significantly increased at the 8-month compared with the 2-month time point but remained constant (LV posterior wall and LV mass) or decreased (LV anterior wall) from 8 months to 14 months (Figure 2, A, B, and E, and Supplemental Figure 3, A and B), while LVID, LV volume, EF, and FS significantly and progressively worsened (Figure 2, C, D, and F, and Supplemental Figure 3, C–E).

The female CUG960 +dox mice showed similar but less severe patterns in all parameters compared to males (Figure 2 and Supplemental Figure 3; significant differences between males and females are marked using purple bars). We also observed expected cardiac aging phenotypes in the MHCrtTA +dox control cohort, including increased heart size and decreased contractility (Figure 2 and Supplemental Figure 3), as described in the literature (34, 39, 40); however, the changes were notably smaller compared with those associated with long-term CUG_exp_ RNA expression in the CUG960 +dox cohort.

Overall, these findings indicate that long-term expression of CUG_exp_ RNA caused progressively enlarged and hypertrophic hearts in the early stage followed by features consistent with cardiac dilation that was associated with impaired contractile function. We also identified sex differences in which male CUG960 +dox mice had more severe disease progression than females.

### Long-term CUG_exp_ RNA expression results in progressive cardiac conduction defects and reduced lifespan.

**Figure 3:**
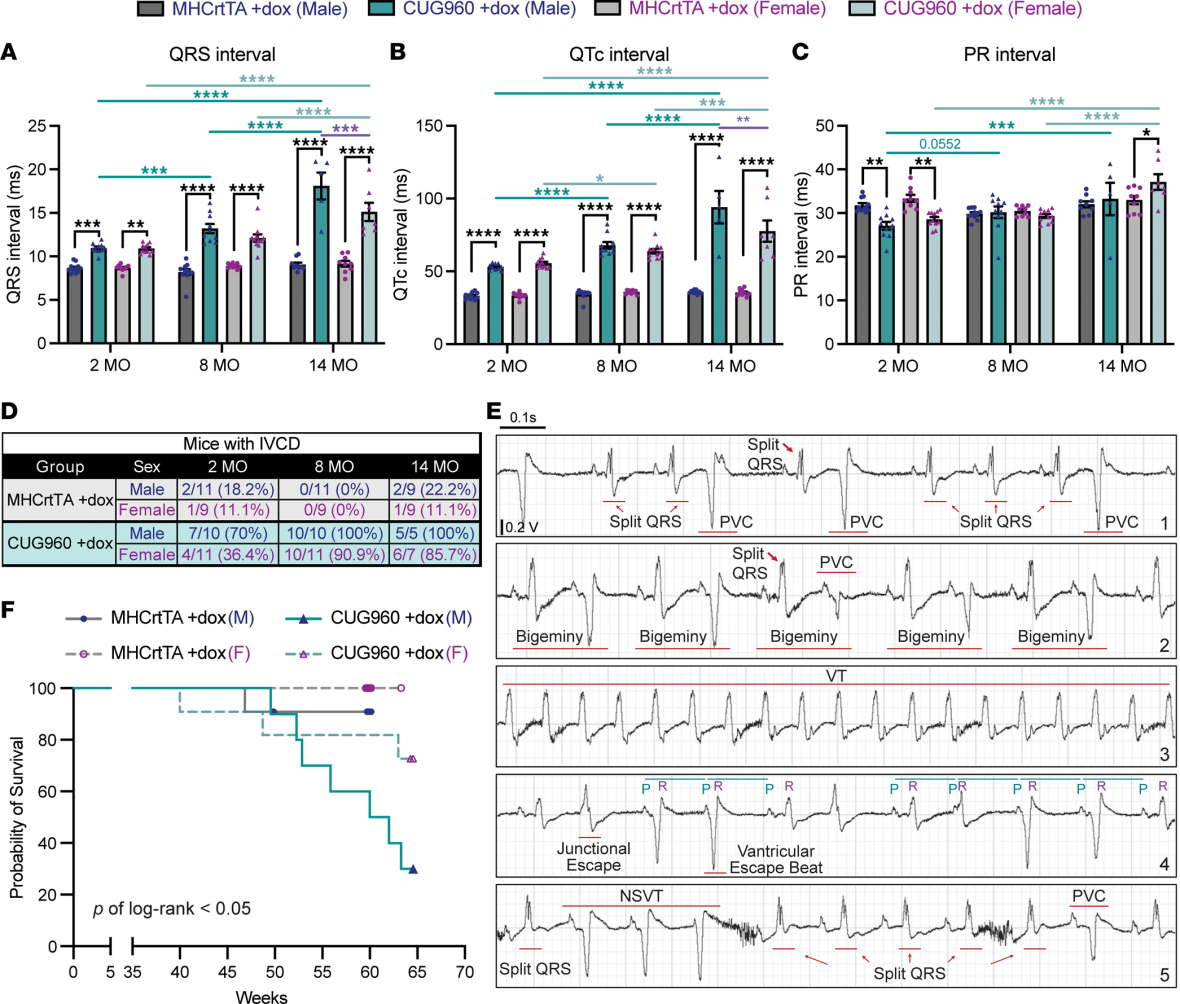
Long-term CUG_exp_ RNA expression resulted in progressive prolongation of cardiac conduction intervals and increased prevalence of abnormal cardiac rhythms. Prospective measurements of (**A**) QRS, (**B**) QTc, and (**C**) PR intervals were determined by surface ECG recordings in CUG960 +dox and MHCrtTA +dox control mice at 2, 8, 14 months of age. Data represent the mean ± SEM and were analyzed using 2-way ANOVA followed by Tukey’s multiple-comparison test. *P < 0.05; **P < 0.01; ***P < 0.001; ****P < 0.0001. (**D**) Numbers of animals per sex showing intraventricular conduction delay (IVCD) on surface ECG tracings. (**E**) Representative ECG tracings showing arrhythmias frequently observed in the CUG960 +dox mice. Split QRS complex indicating bundle branch block and IVCD was observed in all mice shown in panels 1–5. Abnormalities include premature ventricular contraction (PVC; panels 1 and 2), sustained ventricular tachycardia (VT; panel 3), third-degree atrioventricular block (complete heart block; panel 4), and non-sustained ventricular tachycardia (NSVT; panel 5). Images were taken from lead I (left arm to right arm) after high-pass filter (cutoff frequency: 5 Hz) were applied. (**F**) Kaplan-Meier survival analysis showed a reduced lifespan in CUG960 +dox mice, especially in male mice with CUG_exp_ RNA expression. The log-rank (Mantel-Cox) test showed that survival curves were significantly different, with a *P* value of 0.0299. MHCrtTA+dox cohort: *n* = 20 (11 males and 9 females). CUG960 +dox cohort: *n* = 21 (10 males and 11 females). Males (blue symbols) and females (purple symbols) are indicated.

Consistent with our previous studies (32, 33), CUG960 +dox mice showed prolongation of QRS and QTc intervals compared with the MHCrtTA +dox controls at all 3 time points (Figure 3, A and B). Prolonged PR intervals are characteristic of DM1 conduction delays (18, 19). However, this characteristic has not been reproduced with relatively short-term CUG_exp_ RNA expression in mouse heart models (32, 33, 41). PR intervals of CUG960 +dox mice were significantly shorter than the MHCrtTA +dox control animals at 2 months, as observed previously (32), and became significantly prolonged at 14 months (Figure 3C). Importantly, QRS, QTc, and PR intervals progressively increased in CUG960 +dox mice, but not the MHCrtTA +dox control mice (Figure 3, A–C). Moreover, the increased QRS and QTc intervals with 14-month CUG_exp_ RNA expression were significantly longer in males than females (Figure 3, A and B).

In addition, the majority of CUG960 +dox mice displayed intraventricular conduction delay (IVCD), characterized by prolonged conduction intervals and/or BBB, indicating asynchronous ventricular depolarization (details in Methods; Figure 3D and Supplemental Data File 1). The prevalence of IVCDs also significantly increased throughout long-term CUG_exp_ RNA expression in CUG960 +dox mice (Figure 3D; 11/21, 20/21 and 11/12 in CUG960 +dox mice vs. 3/20, 0/20, and 3/18 in MHCrtTA +dox controls at 2, 8, and 14 months, respectively). In addition to IVCD, several types of cardiac arrhythmias in CUG960 +dox mice were first detected at 8 months of age and with increased prevalence at 14 months, especially in males (Supplemental Figure 4 and Supplemental Data File 1). The observed arrhythmias included premature ventricular contraction (PVC), premature atrial contraction (PAC), sustained ventricular tachycardia (VT), AV block, and non-sustained ventricular tachycardia (NSVT) (Figure 3E and Supplemental Figure 4). In Figure 3E, split QRS complexes indicating IVCD are illustrated in all panels. Panels 1 and 2 show different types of PVCs that were often observed in 8- and 14-month-old CUG960 +dox mice (including bigeminy, defined as every normal heartbeat followed by a premature ventricular beat). Panel 3 demonstrates tachycardia with a wide QRS and no P wave, consistent with sustained VT. Panel 4 shows abnormal interval patterns consistent with third-degree AV block (complete heart block), including varying PR intervals yet regular PP intervals, and different QRS morphologies that could be attributed to QRS arising from junctional escape beat or ventricular escape beat. Panel 5 presents NSVT (Figure 3E).

CUG960 +dox mice with long-term CUG_exp_ expression showed an increased prevalence of cardiac electrophysiological disorders (IVCD and/or arrhythmias; details in Methods) over time. Examples of electrophysiological disorder progression in CUG960 +dox compared with control MHCrtTA +dox mice are shown in Supplemental Figure 4 and ECG evaluations for all mice at the 3 time points are provided in Supplemental Data File 1. While MHCrtTA +dox mice showed normal ECG patterns at all 3 time points, male CUG960 +dox mouse Y668 showed IVCD with a wide S wave and a split QRS complex in leads I and II starting at 2 months of age, which progressed to other leads and developed worsening QRS prolongation and bigeminy PVC leading to sustained VT by 14 months (Supplemental Figure 4, A–C, and Supplemental Data File 1). Male Y669 presented with normal sinus rhythm at 2 months of age, had IVCD with a split QRS and frequent PACs at 8 months, and developed worsening QRS prolongation with NSVT by 14 months (Supplemental Figure 4D and Supplemental Data File 1). Female Y739 showed normal sinus rhythm, and female Y742 showed IVCD with split QRS at 2 months; both female mice presented with split QRS complexes at 8 months of age and developed frequent PVCs and NSVT by 14 months (Supplemental Figure 4, E and F, and Supplemental Data File 1).

In addition, 14-month-old male CUG960 +dox mice showed low EF, suggesting potential heart failure (Figure 2H). The observation of electrophysiological disorders and low EF with long-term CUG_exp_ RNA expression correlated with reduced lifespan, particularly in males (Figure 3F). Taken together, progressive conduction delays and increased frequency and severity of cardiac electrophysiological disorders were identified in CUG960 +dox mice throughout long-term CUG_exp_ RNA expression. Moreover, the observed abnormal cardiac rhythm and low contractility resulted in reduced lifespan, particularly in males, similar to the sex differences in cardiac involvement reported in DM1 (14, 17, 20).

### Misregulated splicing did not worsen with long-term CUG_exp_ RNA expression.

**Figure 4:**
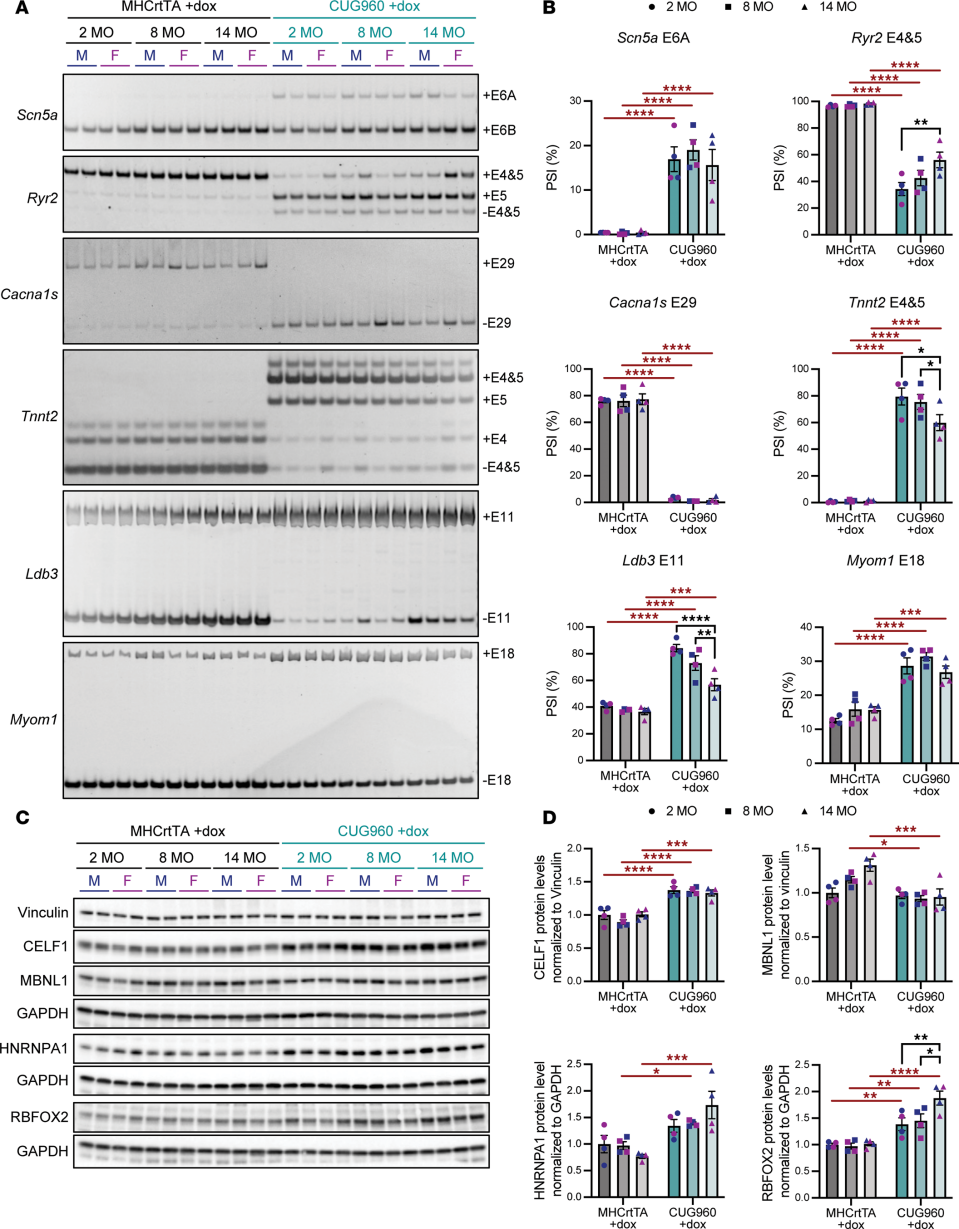
Splicing abnormality and most of misregulated RNA-binding proteins tested did not show progression with long-term CUG_exp_ RNA expression. (**A**) Representative RT-PCR results showing alternative splicing events in left ventricles of CUG960 +dox and control MHCrtTA +dox mice at 2, 8, and 14 months of CUG_exp_ RNA expression. (**B**) Quantitation of percentage spliced in (PSI) of *Scn5a* exon 6A (E6A), *Ryr2* E4&5, *Cacna1s* E29, *Tnnt2* E4&5, *Ldb3* E11, and *Myom1* E18 in ventricular tissues of CUG960 +dox and MHCrtTA +dox mice at different time points. (**C**) Representative Western blot images of CELF1, MBNL1, HNRNPA1, RBFOX2, GAPDH, and VINCULIN from left ventricular protein extracts. (**D**) Quantitation of each protein normalized to VINCULIN or GAPDH. *n* = 4 animals per cohort. Males (2, blue symbols) and females (2, purple symbols) are indicated. Data represent the mean ± SEM and were analyzed using 2-way ANOVA followed by Tukey’s multiple-comparison test. *P < 0.05; **P < 0.01; ***P < 0.001; ****P < 0.0001.

DM1 is characterized by misregulated splicing due to the effect of CUG_exp_ RNA on the activities of RBPs, primarily MBNL sequestration and loss of function (5, 42). The CUG960 model reproduces misregulated splicing observed in DM1 heart tissues (32, 33). To determine whether the disrupted splicing patterns show increased severity that correlate with progressive cardiac defects caused by long-term CUG_exp_ RNA expression, 6 MBNL-dependent splicing events that are misregulated in DM1 heart tissues and the CUG960 DM1 heart mouse model were assayed for changes over the time course (32, 33, 43, 44). Due to the progressive conduction, contractility, and structural defects identified, we assayed splicing events encoding proteins that participate in ion transport (*Scn5a*, *Ryr2*, and *Cacna1s*) and sarcomere structure (*Tnnt2*, *Ldb3*, and *Myom1*). Robust misregulated splicing consistent with DM1 was observed for all 6 genes in LV tissues of CUG960 +dox mice at all 3 time points; however, we did not observe worsening of the misregulation at longer time points (Figure 4, A and B). In fact, the extent of the mis-splicing patterns for *Ryr2* exon 4 and 5 (E4+5), *Tnnt2* E4+5, and *Ldb3* E11 was significantly less in left ventricular samples with 14 months of CUG_exp_ RNA expression compared with earlier time points, while *Scn5a* E6A, *Cacna1s* E29, and *Myom1* E18 splicing misregulation did not change with long periods of CUG_exp_ RNA expression (Figure 4B). While not correlated with physiological phenotype progression, the lack of increased misregulated splicing is consistent with a stable CTG repeat that produces a CUG_exp_ RNA with a constant level of MBNL sequestration and loss of function, indicating that disease progression does not require increased MBNL loss of function (see Discussion).

To determine whether reduced splicing misregulation could be caused by reduced expression of the CTG repeat transgene, we measured CUG_exp_ RNA levels by quantitative reverse transcription PCR (RT-qPCR) analysis in CUG960 +dox LV tissues from each time point, as well as the level of rtTA protein by Western blotting. We found significant decreases in both CUG_exp_ RNA and rtTA protein expression in the 14-month- compared with the 2-month-expression cohort (Supplemental Figure 5, A and B), suggesting that the decreased CUG_exp_ RNA expression could be leading to a slight reversal of sensitive splicing events (see Discussion).

### Expression of RBPs associated with DM1 pathogenesis is altered by CUG_exp_ RNA expression.

Several RBPs in addition to MBNL have been proposed to contribute to or modify DM1 disease severity in skeletal muscle and heart (45–47). To test the effects of long-term CUG_exp_ RNA expression on levels of several RBPs, we isolated proteins from LV tissues of CUG960 +dox and MHCrtTA +dox cohorts that had been on dox for 2, 8, and 14 months. Similar to what has been reported in DM1 (42, 45, 46, 48, 49), protein levels of MBNL1 were significantly decreased, while those of CELF1, HNRNPA1, and RBFOX2 were significantly increased in CUG960 +dox mice compared with the MHCrtTA +dox cohort (Figure 4, C and D). However, altered protein levels of MBNL1, CELF1, and HNRNPA1 did not significantly change during prolonged CUG_exp_ RNA expression. Interestingly, RBFOX2 protein showed a significant increase between 8 and 14 months (Figure 4, A and B).

Overall, the molecular data showed drastic splicing misregulation and altered RBP expression consistent with DM1; however, in contrast with the progression of physiological phenotypes, splicing misregulation and misregulation of the majority of RBPs were not increased by the long-term CUG_exp_ RNA expression.

### Cardiac structural and conductive defects showed reduced reversibility with long-term CUG_exp_ RNA expression.

**Figure 5:**
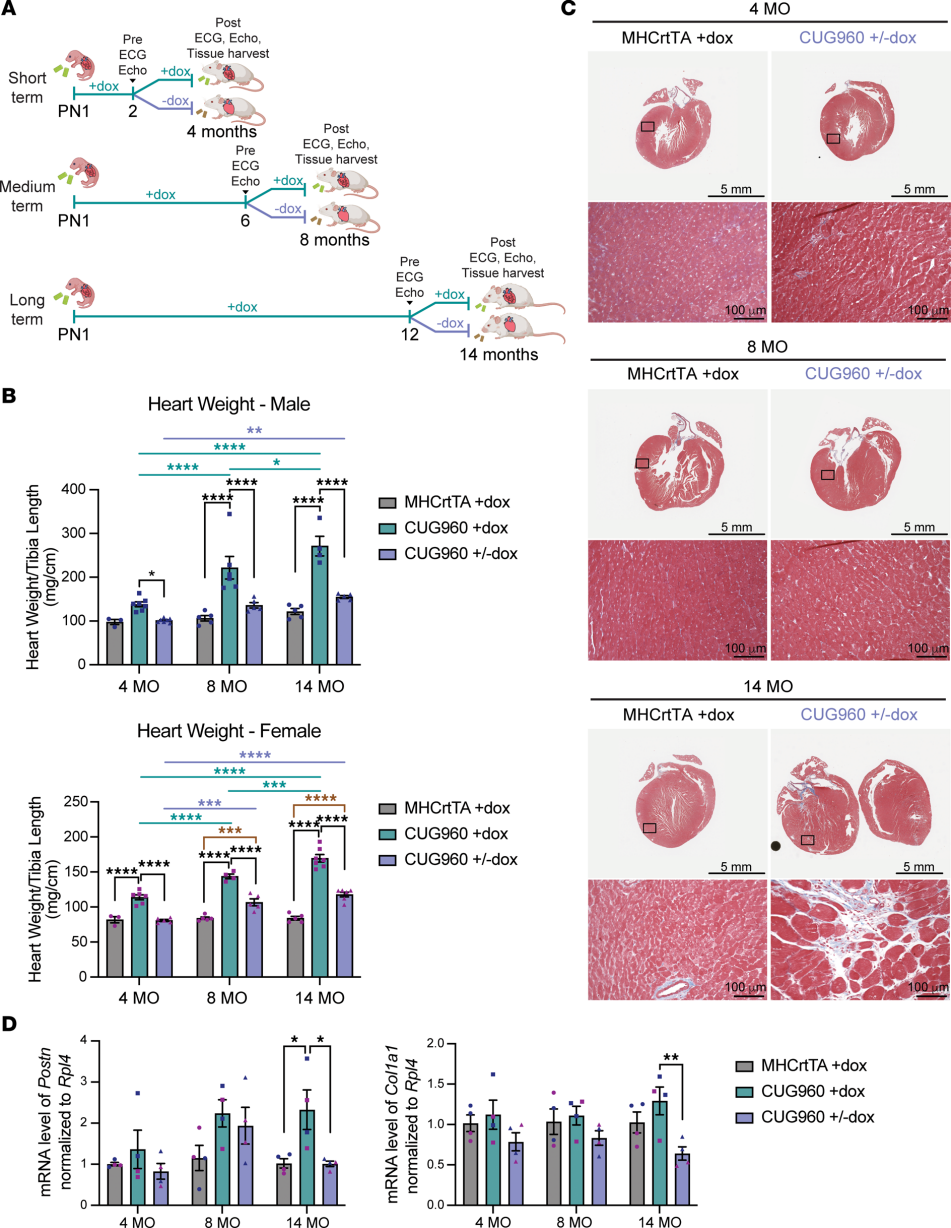
Heart weights and fibrosis marker expression, but not ventricular fibrosis, were rescued after stopping long-term CUG_exp_ RNA expression for 2 months. (**A**) Illustration of the experimental timeline and cohorts. (**B**) Heart weight/tibia length measurements of male and female CUG960 +dox (teal bars) and CUG960 ±dox (purple bars) mice at 4, 8, and 14 months of age. *n* ≥ 4 per time point and sex. (**C**) Representative images of trichrome staining from 4-chamber heart sections of male MHCrtTA +dox control and CUG960 ±dox mice at 4, 8, and 14 months of age. Two animals were examined per sex per group. Section thickness: 4 μm. Scale bars: 5 mm (whole-heart images) and 100 μm (zoomed-in images). (**D**) *Postn* and *Col1a1* mRNA levels in left ventricular tissues of MHCrtTA +dox, CUG960 +dox, and CUG960 ±dox mice at 4, 8, and 14 months of age. Males (blue symbols) and females (purple symbols) are indicated. *n* = 4 per time point (2 males and 2 females). Data represent the mean ± SEM and were analyzed using 2-way ANOVA followed by Tukey’s multiple-comparison test. *P < 0.05; **P < 0.01; ***P < 0.001; ****P < 0.0001.

We showed previously that turning off CUG_exp_ RNA expression after 2 months rescues cardiac defects in the CUG960 heart model to nearly control levels, including heart weight, conduction defects, cardiac morphology, function abnormalities, and misregulated splicing (32, 33). To determine the degree of physiological and molecular rescue after long-term CUG_exp_ RNA expression, we used 3 cohorts: CUG_exp_ RNA expression induced on PN1 for 2 (short-term), 6 (medium-term), and 12 (long-term) months, followed by dox removal (CUG960 ±dox) to turn off CUG_exp_ RNA expression for 2 months (Figure 5A). Two control cohorts were included: CUG960 +dox mice to compare disease phenotypes at corresponding end point ages and MHCrtTA +dox mice without CUG_exp_ RNA expression as a baseline for each end point (Figure 5A). ECG and echo were conducted before animals were taken off dox and 2 months later to determine the level of rescue. Hearts were harvested at each end point for histological and molecular analysis (Figure 5A).

In the short-term cohort, the HW/TL ratio of CUG960 ±dox was significantly rescued to the level of the MHCrtTA +dox control; however, with longer periods of CUG_exp_ RNA expression, CUG960 ±dox female mice in particular retained a higher HW/TL ratio compared with age-matched controls (Figure 5B). As described above, fibrotic tissue was observed after 14 months of CUG_exp_ RNA expression, particularly in males, but not in the 2- and 8-month cohorts (Figure 1E and Supplemental Figure 6). Male CUG960 ±dox mice taken off dox after 12 months of CUG_exp_ RNA expression showed fibrotic tissue in LVs (Figure 5C). In contrast, the expression of fibrosis marker mRNAs in LV tissues of this cohort showed complete rescue (Figure 5D). This observation suggests that while molecular signaling promoting fibrosis was normalized by loss of CUG_exp_ RNA expression, fibrotic tissues could not be cleared within the 2-month timeframe.

**Figure 6:**
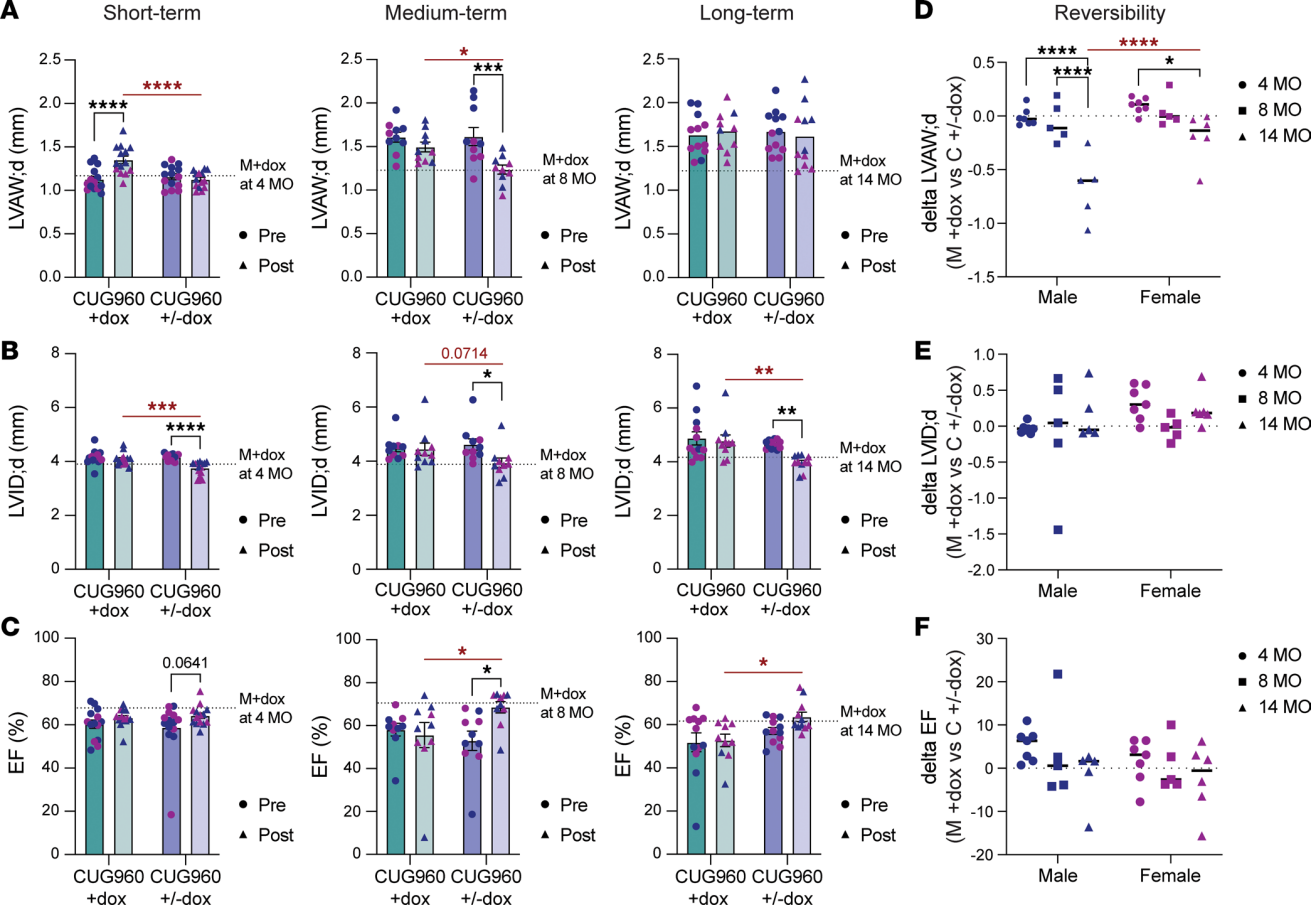
Reversibility of structural parameters are reduced following long-term compared with short-term CUG_exp_ RNA expression. M-mode echocardiography was performed on CUG960 +dox (teal bars) and CUG960 ±dox (purple bars) mice with short-, medium-, and long-term expression of CUG_exp_ RNA. Dark teal and dark purple bars show results from parallel cohorts on dox for short-, medium- or long-term that either remained on dox for 2 additional months (light teal) or were taken off dox for 2 months (light purple). Dark and light teal bars are from the same animals as are dark and light purple bars. (**A**) Left ventricle anterior wall thickness in diastole (LVAW; d), (**B**) LV internal diameter in diastole (LVID; s), and (**C**) ejection fraction (EF) were evaluated. M+dox indicates average values in MHCrtTA +dox controls (not expressing CUG_exp_ RNA) at the age indicated. (**D**–**F**) Reversibility of each parameter was calculated by subtracting values of that parameter from CUG960 ±dox with averaged MHCrtTA +dox control value of the corresponding parameter and time point. *n* ≥ 4 per group, time point, and sex. Data represent the mean ± SEM and were analyzed using 2-way ANOVA followed by Tukey’s multiple-comparison test. *P < 0.05; **P < 0.01; ***P < 0.001; ****P < 0.0001.

M-mode short-axis echo analysis showed that the majority of parameters for cardiac morphology and contractility were rescued after dox removal in short-term and medium-term cohorts, while reversibility was variable in the long-term cohort (Figure 6, A–C, and Supplemental Figure 7). To quantify and compare reversibility between the 3 cohorts, the value of CUG960 ±dox cohort at each time point was subtracted from that of the corresponding MHCrtTA +dox control cohort (Figure 6, D–F, and Supplemental Figure 7). Similar to the sex difference observed for the progression of cardiac defects, male, but not female, CUG960 ±dox mice demonstrated reduced reversibility after dox removal with increased time of CUG_exp_ RNA expression. For example, male CUG960 ±dox mice in the long-term group displayed reduced reversal of cardiac structural parameters, including the LV wall thickness during both systolic and diastolic phases and increased LV mass (Figure 6D and Supplemental Figure 7, I–L). With regard to the variability of rescue, similar effect sizes were observed in the reversibility of the 3 groups for LVID, LV volume, EF, and FS (Figure 6, E and F, and Supplemental Figure 7, M–P).

**Figure 7:**
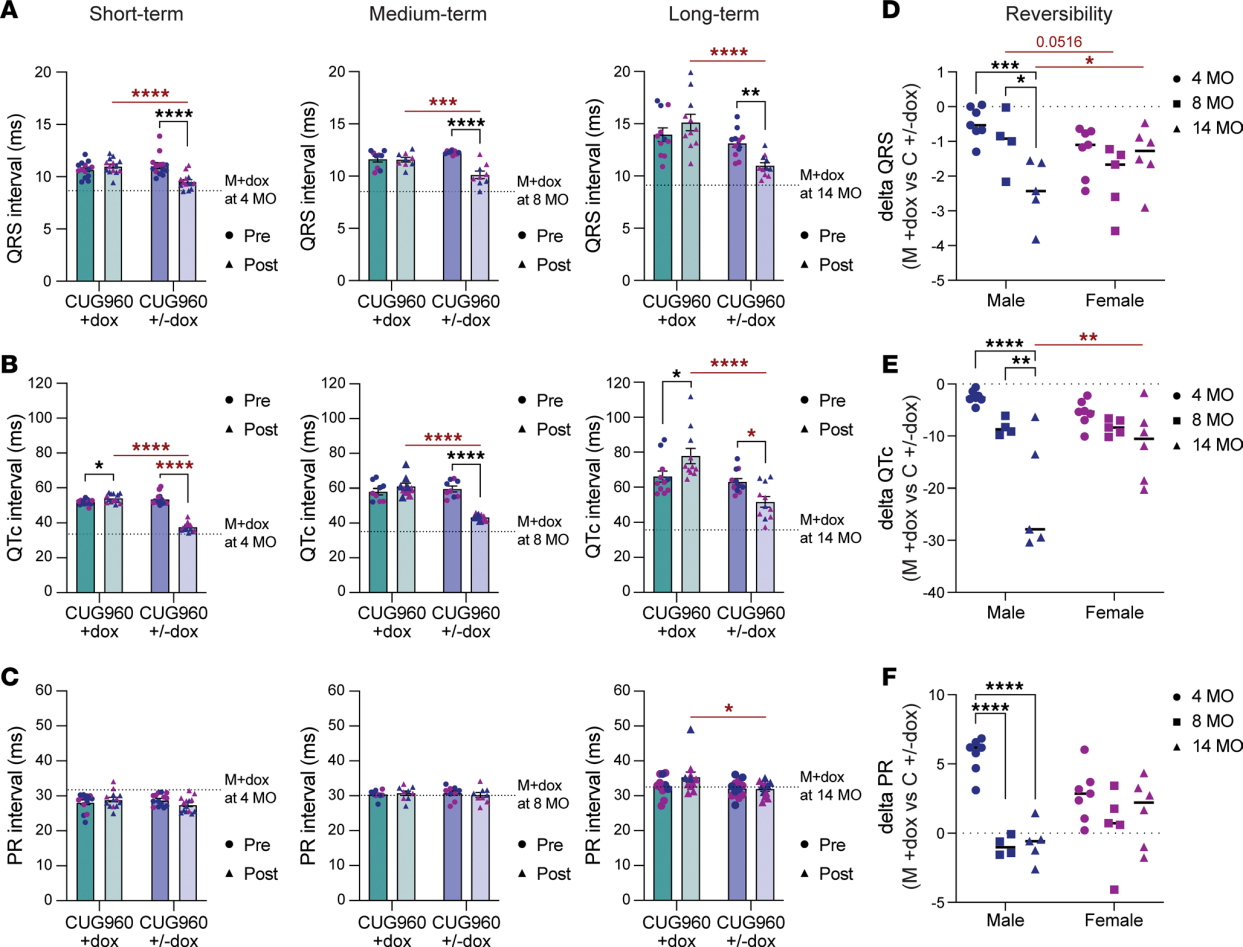
Reduced reversibility of prolonged conduction intervals was observed following long-term CUG_exp_ RNA expression compared with short-term cohorts. (**A**) QRS intervals, (**B**) QTc intervals, and (**C**) PR intervals were determined by surface ECG recordings in CUG960 +dox (teal bars) and CUG960 ±dox (purple bars) mice at short-, medium-, and long-term CUG_exp_ RNA expression. Dark teal and dark purple bars are from parallel cohorts on dox for short-, medium- or long-term that either remained on dox for 2 additional months (light teal) or were taken off dox for 2 months (light purple). Dark and light teal bars are from the same animals as are dark and light purple bars. The dotted lines indicate average of MHCrtTA +dox controls at the age indicated. (**D**–**F**) Reversibility of each parameter was calculated by subtracting the value of CUG960 ±dox from averaged MHCrtTA +dox control value of the corresponding parameter and time point. *n* ≥ 4 per group, time point, and sex. Data represent the mean ± SEM and were analyzed using 2-way ANOVA followed by Tukey’s multiple-comparison test. *P < 0.05; **P < 0.01; ***P < 0.001; ****P < 0.0001.

ECG analysis was also conducted to determine the reversibility of cardiac conduction phenotypes induced by CUG_exp_ RNA expression in the short-, medium-, and long-term cohorts (Figure 5A). We found that the prolonged QRS and QTc intervals of CUG960 ±dox mice were significantly improved for all 3 cohorts, comparing individual CUG960 ±dox mice just before and 2 months after dox removal (Figure 7, A and B). Moreover, the levels of improvement between the same CUG960 ±dox mice before and after dox withdrawal were comparable in short-, medium-, and long-term cohorts. However, in comparison with MHCrtTA +dox controls at corresponding time points to determine reversibility back to baseline controlled for age, male CUG960 ±dox mice with long-term CUG_exp_ RNA expression showed significantly less reversibility of QRS and QTc prolongation than mice in short- and medium-term groups due to disease progression (Figure 7, D and E). In contrast, there was no significant difference observed between female CUG960 ±dox mice at different time points (Figure 7, D and E). Analysis of PR interval changes in the long-term CUG960 ±dox cohort compared with CUG960 +dox mice of the same time point showed significant rescue toward the intervals observed in MHCrtTA +dox control mice. While PR intervals were increased in medium-term and long-term cohorts, we were not able to use the comparison of CUG960 ±dox and MHCrtTA +dox mice to calculate reversibility (Figure 7F) because PR intervals of CUG960 +dox mice were shorter compared with MHCrtTA +dox control at 2 months of age (32) (Figure 3C and Figure 7C).

Moreover, the reversibility of cardiac electrophysiological disorders decreased in CUG960 ±dox mice with long-term CUG_exp_ expression, especially in male animals, compared with the short-term cohort (Supplemental Figure 8 and Supplemental Data File 2). In the short-term cohort, 8 of 14 (3 male and 5 female) CUG960 ±dox mice showed cardiac electrophysiological disorders at 2 months of age before turning off the CUG_exp_ RNA expression. After stopping CUG_exp_ RNA expression for 2 months, 6 of these 8 animals showed a normal ECG pattern. Importantly, reversibility was reduced with the increased periods of CUG_exp_ RNA expression; in the medium-term cohort, 5 (3 males and 2 female) out of the 9 (5 males and 4 females) CUG960 ±dox mice that displayed cardiac electrophysiological disorders before turning off the CUG_exp_ RNA expression showed normal sinus rhythm 2 months after stopping CUG_exp_ RNA expression. Reversibility decreased further in long-term cohorts, in which only 4 (1 male and 3 females) out of the 11 (6 males and 5 females) CUG960 ±dox mice that showed cardiac electrophysiological disorders were rescued after stopping the CUG_exp_ RNA expression (Supplemental Figure 8 and Supplemental Data File 2).

Overall, the structural and electrophysiological cardiac abnormalities were rescued after cessation of CUG_exp_ RNA expression at all time points, but the degrees of reversibility varied, with less reversibility observed with longer periods of CUG_exp_ RNA expression, specifically in conduction delays, increased LV wall thickness and LV mass, as well as fibrotic tissue formation.

### Misregulated splicing but not all misregulated RBPs were rescued to control levels in CUG960 ±dox mice with long-term CUG_exp_ RNA expression.

**Figure 8:**
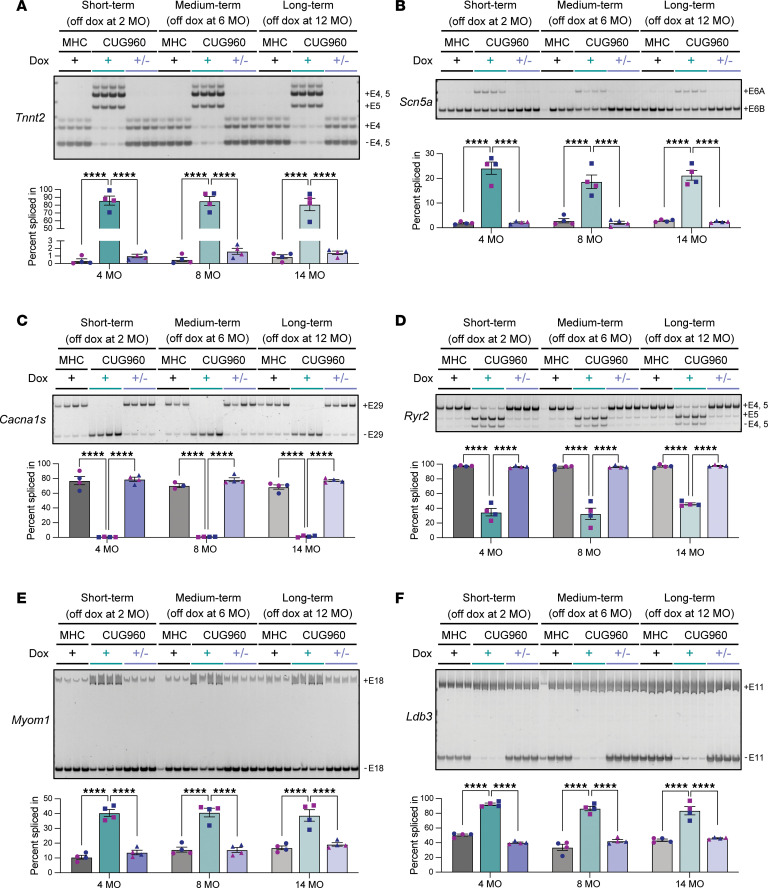
Misregulated splicing events in CUG960 +dox mice returned to control levels in short-, medium-, and long-term cohorts after turning off CUG_exp_ RNA expression. Representative RT-PCR results and quantitation of percentage spliced in (PSI) for (**A**) *Tnnt2* exons 4 and 5 (E4, 5); (**B**) *Scn5a* E6A; (**C**) *Cacna1s* E29; (**D**) *Ryr2* E4, 5; (**E**) *Myom1* E18; and (**F**) *Ldb3* E11 in ventricles of control MHCrtTA +dox (gray bars), CUG960 +dox (teal bars), and ±dox (purple bars) mice at 4, 8, and 14 months of age. *n* = 4 animals per cohort. Males (2, blue symbols) and females (2, purple symbols) are indicated. Data represent the mean ± SEM and were analyzed using 2-way ANOVA followed by Tukey’s multiple-comparison test. ****P < 0.0001.

To determine the extent of rescue for misregulated alternative splicing and altered RBP expression induced by CUG_exp_ RNA at different time points, we looked at the 6 splicing events and 4 RBPs assayed during the time course. RT-PCR analysis of splicing demonstrated that all misregulated splicing events were completely rescued to MHCrtTA +dox levels in all 3 cohorts (Figure 8), in contrast with the incomplete reversibility of cardiac structural and conductive abnormalities. Interestingly, the RBPs that were misregulated in the CUG960 +dox mice demonstrated different levels of reversibility after turning off CUG_exp_ RNA expression. CELF1 upregulation was significantly reversed in CUG960 ±dox of short-, medium-, and long-term cohorts, while the reversibility in the long-term cohort was significantly less than the short-term cohorts (Supplemental Figure 9, A and B). Decreased MBNL protein levels were rescued after turning off CUG_exp_ RNA expression in all 3 cohorts compared with corresponding CUG960 +dox controls (Supplemental Figure 9, A and C). HNRNPA1 and RBFOX2 were both significantly upregulated in CUG960 +dox mice of medium- and long-term cohorts, but these misregulated expression levels displayed different reversibility. While the level of HNRNPA1 significantly decreased after removal of dox in the long-term cohort compared with CUG960 +dox mice, there was no change observed for RBFOX2 protein expression between CUG960 +dox and ±dox mice in both medium- and long-term cohorts, indicating that removal of transgene expression did not rescue RBFOX2 misregulation within the 2-month timeframe (Supplemental Figure 9, A, D, and E).

## Discussion

DM1 manifestations progress throughout the lifetime of affected individuals (20). A main driver of disease progression is likely to be the dramatic increase in CTG repeat number over time due to somatic instability (50–52). The resulting expansion of the CUG repeat track is expected to increase intrinsic toxicity of the *DMPK* mRNA due primarily to increased MBNL sequestration and loss of function. In this study, we used our DM1 heart model to test the physiological and molecular effects of long-term expression of CUG_exp_ RNA containing a stable CUG repeat number. Using the ability to shut off CUG_exp_ RNA expression, we also tested the degree to which cardiac features were reversible after different periods of CUG_exp_ RNA expression.

Our results showed progressive worsening of cardiac phenotypes throughout long-term CUG_exp_ RNA expression, including increased heart size and weight, decreased contractile function, altered cardiac structure, and prolonged cardiac conduction intervals compared with age-matched MHCrtTA +dox controls. Consistent with DM1, most of these physiological phenotypes, as well as cardiac fibrosis and reduced longevity, were more severe in males. Importantly, progression of disease features was primarily driven by CUG_exp_ RNA rather than aging alone, as the age-matched MHCrtTA +dox controls did not exhibit comparable cardiac phenotypes.

At the molecular level, misregulated splicing was observed at the earliest time point tested (2 months) but did not worsen at 8 or 14 months of CUG_exp_ RNA expression. This observation suggests that the level of MBNL loss of function does not change over time and is consistent with a stable number of CUG repeats in *DMPK* transcripts. Based on this observation, we conclude that phenotype progression in the CUG960 model is not due to increasing loss of MBNL function. Potential mechanisms of progression include long-term consequences of MBNL loss of function, additional pathological effects of CUG_exp_ RNA, a general cardiac remodeling response to chronic insult, and reduced ability to compensate for a chronic intrinsic insult during aging. For instance, among the RBPs we examined, CELF1 and RBFOX2 were increased at 2 months, and HNRNPA1 was increased by 8 months. CELF1 and HNRNPA1 maintained the same level of increased expression, while RBFOX2 levels increased further at 14 months. These RBPs also showed different levels of rescue or an absence of rescue at different time points. The results support the potential contributions of long-term misregulation of less explored changes as contributing to disease progression.

A possible contribution to disease progression is the premature aging of cells expressing CUG_exp_ RNA. The degenerative process in DM1 has been proposed to resemble accelerated aging (53); several pathogenic mechanisms supporting this premature aging hypothesis have been associated with DM1 phenotypes, including senescence accumulation (54, 55), telomere shortening (56), alteration of autophagy (57), and mitochondrial dysfunction (58). It would be of interest to determine whether a mechanism of early-onset and accelerated aging contributes to CUG960 cardiac disease progression. Analysis of transcriptome changes between 2, 8, and 14 months of CUG_exp_ RNA expression could highlight genes not previously implicated in DM1 pathogenesis without the complication in human datasets caused by the additional impact of somatic expansion.

We found that the extent of the mis-splicing patterns for some splicing events were significantly weaker in LV samples with 14 months of CUG_exp_ RNA expression compared with earlier time points (Figure 4B). This reduction of mis-splicing with long-term expression and aging has also been seen in HSA^LR^ animals expressing 250 CUG repeats in the 3′ UTR of human skeletal muscle actin older than 6 months, and more so in females. In our case, we observed reduced expression of rtTA, which is driven by the *MYH6* promoter. One possibility could be the MHC isoform shift observed in cardiomyopathy, in which there is a decrease in MYH6 expression and an increase in MYH7 expression (62–65). Decreased expression of the MHCrtTA transgene could be a result in response to cardiomyopathy. Consistent with this possibility, we observed significantly decreased expression of the endogenous *Myh6* mRNA and increased *Myh7* mRNA in 14-month LV samples of CUG960 +dox mice by RT-qPCR, which correlated with the deteriorating cardiac phenotypes (data not shown).

While rescue of physiological features showed variability for different parameters, a common theme was that longer periods of CUG_exp_ RNA expression showed decreased reversibility. This was particularly the case for fibrosis within the LV, observed at 14 months but not 8 months of CUG_exp_ expression. Myocardial fibrosis has been shown to not only lead to the abnormalities in cardiac conduction but also work as a substrate for supraventricular and ventricular arrhythmias (66). Fibrosis may also have a central role in the development of the systolic dysfunction observed in patients with DM1 (12, 36, 67, 68). Given that CUG_exp_ RNA was turned off for only 2 months, it remains unclear whether the fibrosis observed in the CUG960 model would show some degree of reversibility following a longer time course without CUG_exp_ RNA.

We note that CUG_exp_ RNA is overexpressed in CUG960 mice compared with the endogenous *Dmpk*. In addition, the CUG repeats are interrupted to maintain stability. Our previous studies and results from several labs having used these repeats support a pathogenic effect representative of DM1. While we have no evidence of different effects compared with pure CUG repeats, it is possible that the interruptions could affect the impact of RNA structure or binding of other factors.

In conclusion, our data demonstrate that the severity of DM1 cardiac conduction, contractility, and structure progress throughout the long-term CUG_exp_ RNA expression in our DM1 heart mouse model. Based on the lack of progressive splicing misregulation indicative of MBNL activity, we conclude that there is a mechanism for disease progression independent of increased MBNL sequestration and loss of function. Our findings underscore the potential benefits of early intervention in patients with DM1 to prevent cardiac disease, thereby potentially averting life-threatening arrhythmias, heart failure, and premature mortality.

## Methods

### Sex as a biological variable.

Our study included both male and female animals, and different findings between sexes are reported, with males having more severe phenotypes.

### Mouse husbandry and transgenic mice.

All animals were housed and maintained on a 12-hour light/dark cycle in the animal facilities at Baylor College of Medicine.

The TREDT960I/TREDT960I; MHC-rtTA (CUG960) mice in the FVB strain background were previously established (32). The TREDT960I mice (stock 032050) are available at The Jackson Laboratory. The MHC-rtTA transgenic mice (FVB/N-Tg(Myh6-rtTA)8585Jam/Mmmh; RRID: MMRRC_010478-MU) were originally acquired from Mutant Mouse Resource & Research Centers (MMRRC) (69). Southern blot analysis of tail DNA from TREDT960I animals after more than 20 generations produced a single band of the expected size for containing the interrupted 960 CTG repeats as in the original transgene plasmid (data not shown).

To generate experimental CUG960 mice, TREDT960I/TREDT960I; MHC-rtTA/MHC-rtTA animals were crossed with TREDT960I/TREDT960I mice. For generating the MHCrtTA hemizygous control mice, we crossed the MHCrtTA hemizygous mice with FVB wild-type animals. Dox-containing chow (2g dox/kg chow, Bioserv) was provided beginning on PN1 through nursing dams. We established this protocol for inducing CUG_exp_ RNA in heart to balance a consistent and robust phenotype obtained with induction at a young age with preventing adverse effects of dox on prenatal development (70, 71). Genomic DNA was extracted from tail clips using DirectPCR lysis reagent (Viagen Biotech) and genotyped by PCR with transgene-specific primers (obtained from Sigma-Aldrich; Supplemental Table 1). The expected amplicon sizes were 331 bp for the TREDT960I allele and 265 bp for MHC-rtTA allele.

### RNA extraction, RT-qPCR, and RT-PCR.

Total RNA was isolated from LV tissues using TRIzol reagent (Invitrogen, 15596018) in a Bullet Blender 2.4 Tissue Homogenizer (Next Advance) with 0.1 g of 0.5 mm diameter Zirconium Oxide Beads (Next Advance). RNA concentrations were measured using the Epoch Microplate Spectrophotometer (BioTek). Random-primed cDNA was prepared from 1 μg of total RNA using a High-Capacity cDNA Reverse Transcription Kit (Applied Biosystems, 4368813).

RT-qPCRs were performed using PowerUp SYBR-Green PCR master mix (Applied Biosystems) and run on the CFX Opus 96 Real-time PCR system (Bio-Rad). All samples were analyzed in triplicate and the expression of levels of *DMPK*, *Postn*, and *Col1a1* were normalized to expression of *Rpl4*. Relative expression levels were determined by the 2^–ΔΔCt^ method. Primers for *DMPK*, *Postn*, *Col1a1*, and *Rpl4* were obtained from Sigma-Aldrich (Supplemental Table 2).

RT-PCR was conducted on cDNA using AmfiSure PCR Master Mix (GenDepot) with designed primers annealing to flanking constitutive exons obtained from Sigma-Aldrich (Supplemental Table 3). PCR products were run in 5% polyacrylamide gels (0.1 M Tris, 0.1 M boric acid, 2 mM EDTA, 5% acrylamide/bis 19:1 [Bio-Rad], 0.1% APS, and 0.18% TEMED [Bio-Rad]) in TBE running buffer (0.1 M Tris, 0.1 M boric acid, 2 mM EDTA). The gels were incubated in 0.4 μg/mL ethidium bromide in TBE for 10 minutes, then imaged using a Molecular Imager ChemiDoc XRS+ with Image Lab software (Bio-Rad). Percentage spliced in (PSI) values were calculated using densitometry according to the following equation: PSI = 100 × (inclusion band/[inclusion band + skipping band]).

### Protein extraction and Western blot from mouse tissues.

Protein extracts were prepared from LV tissues by homogenization in 1× RIPA lysis buffer (Cell Signaling Technology, 9806) supplemented with 1× Halt protease and phosphatase inhibitor cocktail (Thermo Fisher Scientific, 78440) and 0.10 g of 1.0 mm diameter Zirconium Oxide Beads (Next Advance) using the Bullet Blender 2.4 Tissue Homogenizer (Next Advance) followed by sonication. Cellular debris was removed by centrifugation at 18,000*g* for 15 minutes at 4°C. Protein concentrations in the supernatant were measured using the Pierce BCA Protein Assay Kit (Thermo Fisher Scientific). Samples were diluted in 6× Laemmli SDS sample buffer (Alfa Aesar, J61337), boiled for 5 minutes, and 30 μg of total protein per sample was run in 4%–20% Tris-glycine SDS-PAGE gels (Bio-Rad, 5678095) using Tris/Glycine/SDS Running Buffer (Bio-Rad).

Proteins were subsequently transferred to Trans-Blot Turbo Nitrocellulose membranes using the Trans-Blot Turbo system at 2.5 A and 25 V for 7 minutes in Trans-Blot Turbo Transfer Buffer (all Bio-Rad). Membranes were washed in phosphate-buffered saline (PBS; 10 mM Na_2_HPO_4_, 2.7 mM KCl, 137 mM NaCl, 1.76 mM KH_2_PO_4_, pH 7.4) containing 0.01% Tween 20 (Sigma-Aldrich) (PBST) for 5 minutes and blocked for 40 minutes at room temperature in 5% Blotto Non-Fat Dry-Milk (Chem Cruz) prepared in PBST. Membranes were then incubated overnight at 4°C with primary antibodies against MBNL1 (in-house antibody made by Bethyl Laboratories; 1:4,000 dilution), CELF1 (Millipore, 05-621, HRP-conjugated in house with HRP conjugation kit from Abcam, 274112; 1:5,000 dilution), HNRNPA1 (Santa Cruz Biotechnology, sc-32301 HRP; 1:2,000 dilution), RBFOX2 (Recombinant Protein Production and Characterization Core, Baylor College of Medicine; 1:4,000 dilution), GAPDH (Cell Signaling Technology, 2118L; 1:10,000 dilution), or vinculin (Sigma-Aldrich, V9131; 1:5,000 dilution) diluted in PBST.

Membranes were washed 3 times in PBST and incubated for 1 hour at room temperature in HRP-conjugated goat anti-rabbit or goat anti-mouse secondary antibodies (Jackson ImmunoResearch; 1:10,000 dilution) diluted in 1% nonfat milk in PBST. After washing 3 times in PBST, membranes were incubated in SuperSignal West Femto Maximum Sensitivity Substrate (Thermo Fisher Scientific, PI34096) for 3 minutes and then imaged on a ChemiDoc XRS+ Imaging system (Bio-Rad).

### Histology.

Mouse hearts were collected and perfused through the aorta with 1× PBS, then fixed in 10% neutral buffered formalin for 48–72 hours. Fixed tissues were submitted to the Histology Laboratory in Pathology department at Baylor College of Medicine for tissue processing and paraffin embedding. Paraffin blocks were subsequently sent to Histowiz Inc. for Masson’s trichrome staining on 4-μm sagittal sections displaying all 4 cardiac chambers. Whole-slide bright-field images were scanned at Histowiz, and higher-magnification images were acquired using an Olympus BX41 microscope with 20× objective lens.

### Echo.

In vivo cardiac function and morphology were evaluated using a Vevo F2 ultrasound machine equipped with a 40 MHz transducer (UHF57x, FujiFilm Visualsonics). Two days before conducting echo, mice were anesthetized with 2% isoflurane and 1.5% oxygen for chest hair removal. For echo recording, mice were under 2% isoflurane while being placed on the heated Rodent Surgical Monitor+ platform (Indus Instruments) and the body temperature was monitored using a rectal probe and LabChart software (AD Instruments) and maintained between 36.5°C and 37.5°C. M-mode images were acquired in the short-axis position at the level of papillary muscles for each animal. Two M-mode tracings were analyzed per animal using the Visualsonics VevoLab analysis package and values were averaged.

### ECG.

ECG data were collected using a multichannel amplifier subsequently converted into digital signals for analysis. Surface ECG recordings were obtained with the Rodent Surgical Monitor+ platform, and data acquisition was performed using PowerLab software (AD Instruments). Mice were anesthetized in an induction chamber with 2% isoflurane and were kept under the same condition for data collection with the Rodent Surgical Monitor+ platform. Body temperature was continuously monitored with a rectal probe connected to LabChart software and maintained between 36.5 and 37.5°C. Data were recorded for 1.5 to 2 minutes per mouse and analyzed using the LabChart software. For one animal, 20 beats were randomly picked to measure the intervals and obtain the averaged values of PR, QRS, and QTc intervals. The QTc interval was calculated according to Bazett’s formula (72). ECG tracings were evaluated by cardiologists to identify cardiac electrophysiological disorders. Images of the ECG tracings were obtained from lead I (left arm to right arm) and II (left leg to right arm) after a high-pass filter (cutoff frequency: 5 Hz) was applied to remove low-frequency artifacts and prevent base shift.

The term “cardiac electrophysiologic disorders” includes all electrophysiologic abnormalities observed, including IVCD and arrhythmias. IVCD is defined as QRS widening and/or abnormal ventricular depolarization morphology. This definition not only includes the QRS prolongation, but also takes the abnormal morphology of the ventricular depolarization (especially split QRS) into account. Under this definition, we were looking for animals with (a) complete BBB (QRS widening with abnormal ventricular depolarization morphology), (b) incomplete BBB (abnormal ventricular depolarization morphology without QRS widening), and (c) nonspecific IVCD (QRS widening without abnormal ventricular depolarization that meets criteria for BBB) (73).

### Statistics.

All quantitative experiments had at least 3 independent biological replicates. Results are presented as mean ± SEM. Statistical analyses for data sets were carried out using Prism software (version 9.0, GraphPad) and methods used are specified in figure legends. Statistical tests for multiple comparisons included 1-way or 2-way ANOVA followed by Tukey’s test. The ROUT method was used to identify outliers. A *P* value of less than 0.05 was considered significant.

### Study approval.

All mouse experiments were carried out in accordance with the NIH *Guide for the Care and Use of Laboratory Animals* (National Academies Press, 2011) and approved by the Baylor College of Medicine Institutional Animal Care and Use Committee.

### Data availability.

Data are available in the main text or the supplemental materials. Values for all data points in the graphs are reported in the supplemental Supporting Data Values file.

## Author contributions

RCH and TAC conceived the study, designed experiments, analyzed and interpreted data. RCH performed the assays and wrote the manuscript. RCH and TAC edited the manuscript. MT and XHTW evaluated the electrocardiogram data and identified abnormal cardiac rhythms. All authors reviewed the manuscript and provided input.

## Conflict of interest

The authors have declared that no conflict of interest exists.

## Funding support

- Muscular Dystrophy Association grant 276796 (to TAC).
- NIH grants R01HL147020 and R01AR082852 (to TAC).
- NIH grants R01HL153350, R01HL160992, R01HL174510, and R01HL180477 (to XHTW).
- Myotonic Dystrophy Foundation predoctoral fellowship (to RCH).
- American Heart Association predoctoral fellowship 23PRE1020500 (to RCH).
- NIH grants S10OD032380, UM1HG006348, and R01DK114356 (partial support for the Baylor College of Medicine Mouse Phenotyping Core).

## Supplementary Material

Supplemental data

Supplemental data set 1

Supplemental data set 2

Unedited blot and gel images

Supporting data values

## Version 1 03/19/2026

In-Press Preview

## Version 2 05/08/2026

Electronic publication
