# Supplementary material for: Progressive cardiac phenotypes and reduced reversibility from long-term CUGexp RNA expression in a DM1 mouse model
Source: JCI Insight. 2026 Mar 19;11(9):e204278. doi: 10.1172/jci.insight.204278 (PMC13232024; doi:10.1172/jci.insight.204278)
Supplement: Supplemental data [file jciinsight-11-204278-s109.pdf]

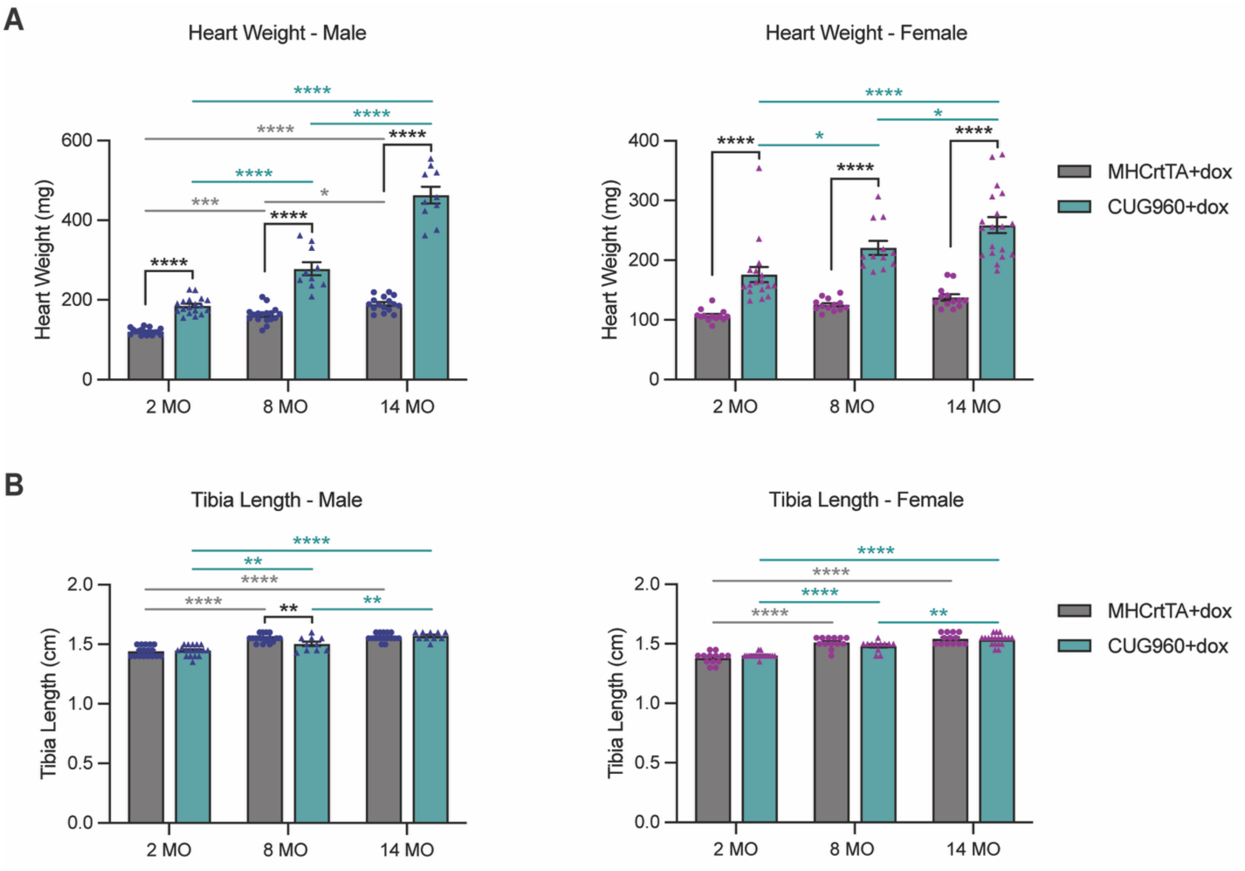

C

Male			
		MHCrtTA +dox	CUG960 +dox
HW/TL (Mean ± SD)	2 MO	83.94 ± 5.88	128.02 ± 11.73
	8 MO	104.93 ± 12.88	185.34 ± 35.00
	14 MO	121.73 ± 12.04	295.04 ± 42.76
% increase	8 vs 2 MO	25.0	44.8
	14 vs 2 MO	45.0	130.4

D

Female			
		MHCrtTA +dox	CUG960 +dox
HW/TL (Mean ± SD)	2 MO	77.64 ± 6.49	125.08 ± 35.42
	8 MO	82.88 ± 5.94	148.53 ± 24.86
	14 MO	89.12 ± 10.47	168.04 ± 35.00
% increase	8 vs 2 MO	6.8	18.8
	14 vs 2 MO	14.8	34.4

Supplemental Figure 1. Increased heart weight/tibia length in CUG960 +dox mice was more profound compared to the MHCrtTA +dox control mice. The **(A)** heart weight (HW) and **(B)** tibia length (TL) of male (left) and female (right) CUG960 +dox and MHCrtTA +dox mice at 2, 8, and 14 months of age (n ≥ 10 per timepoint and sex). **(C and D)** The average of HW/TL for each group and timepoint were used to calculate the percent increase of HW/TL in 8 or 14 months compared to 2 months of age. Data represent the mean ± SEM and were analyzed using two-way ANOVA followed by Tukey's multiple comparisons test. *p<0.05, **p<0.01, ***p<0.001, ****p<0.0001.

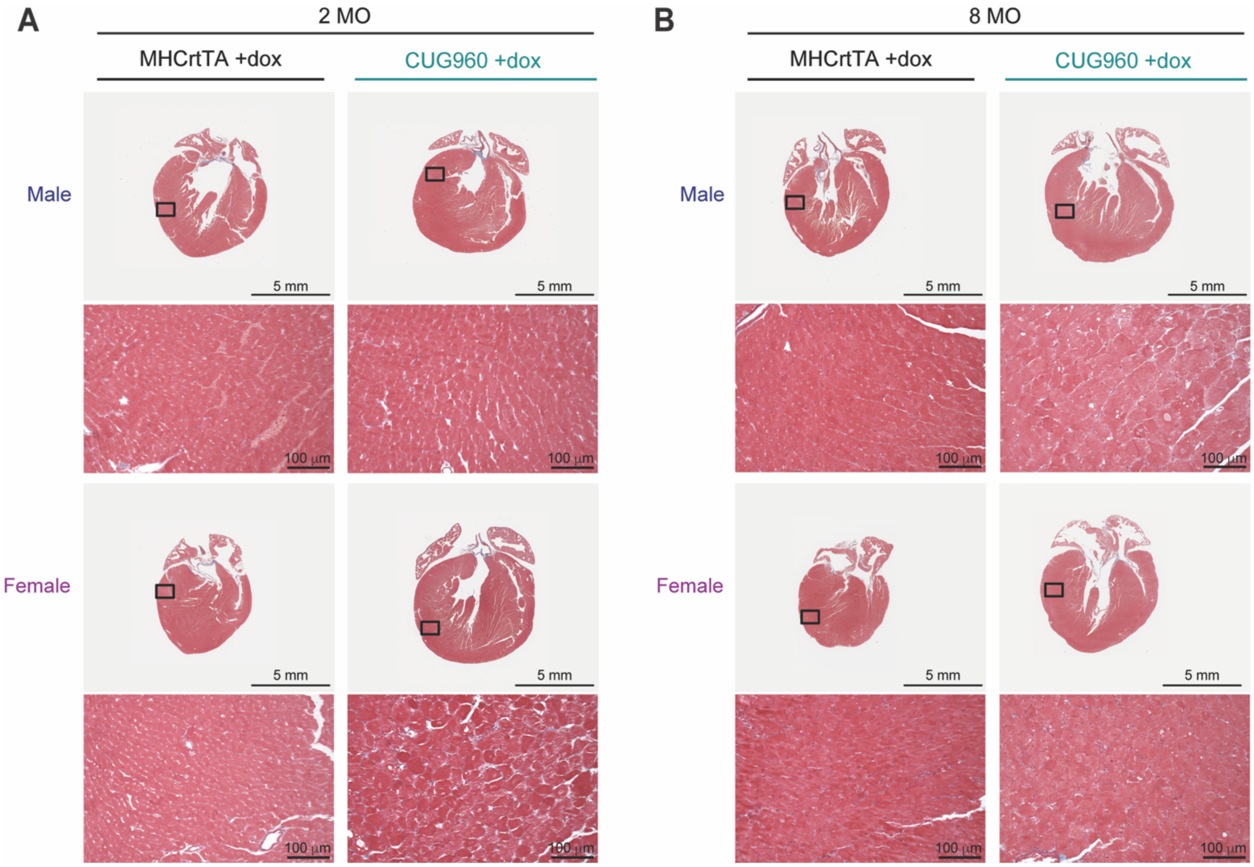

Supplemental Figure 2. Absence of fibrosis in hearts from CUG960 +dox mice after 2 or 8 months of CUG_{exp} RNA expression. Representative images of trichrome staining from four-chamber heart sections of CUG960 +dox and MHCrtTA +dox mice at **(A)** 2 months and **(B)** 8 months of age. Three animals were examined for each group per sex and timepoint. Section thickness: 4 microns. Scale bar for the whole-heart images is 5 mm and for the zoomed-in images is 100 μm.

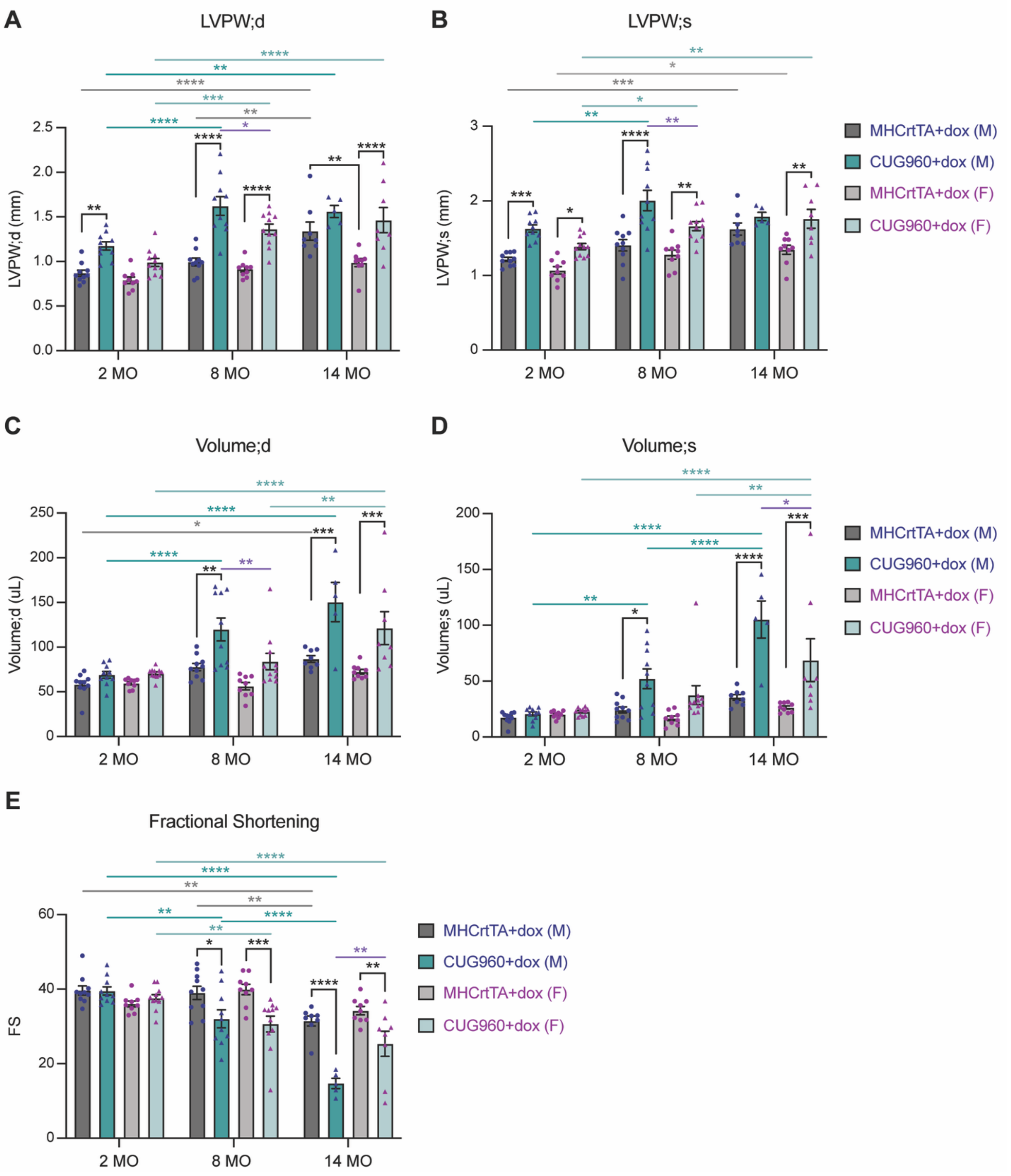

Supplemental Figure 3. CUG960 +dox mice developed progressive structural abnormalities and impaired contractile function with long-term CUG$_{exp}$ RNA expression M-mode echocardiography was performed on the same CUG960 +dox and MHCrtTA +dox mice at 2, 8, and 14 months of age to evaluate: **(A)** left ventricle posterior wall thickness in diastole (LVPW; d, left), **(B)** LVPW in systole (LVPW; s, right), **(C)** volume in diastole, **(D)** volume in systole, and **(E)** fractional shortening (FS). MHCrtTA +dox: 10 males and 9 females. CUG960 +dox: 10 males

and 11 females. Data present the mean ± SEM and were analyzed using two-way ANOVA followed by Tukey's multiple comparisons test. *$p<0.05$, **$p<0.01$, ***$p<0.001$, ****$p<0.0001$.

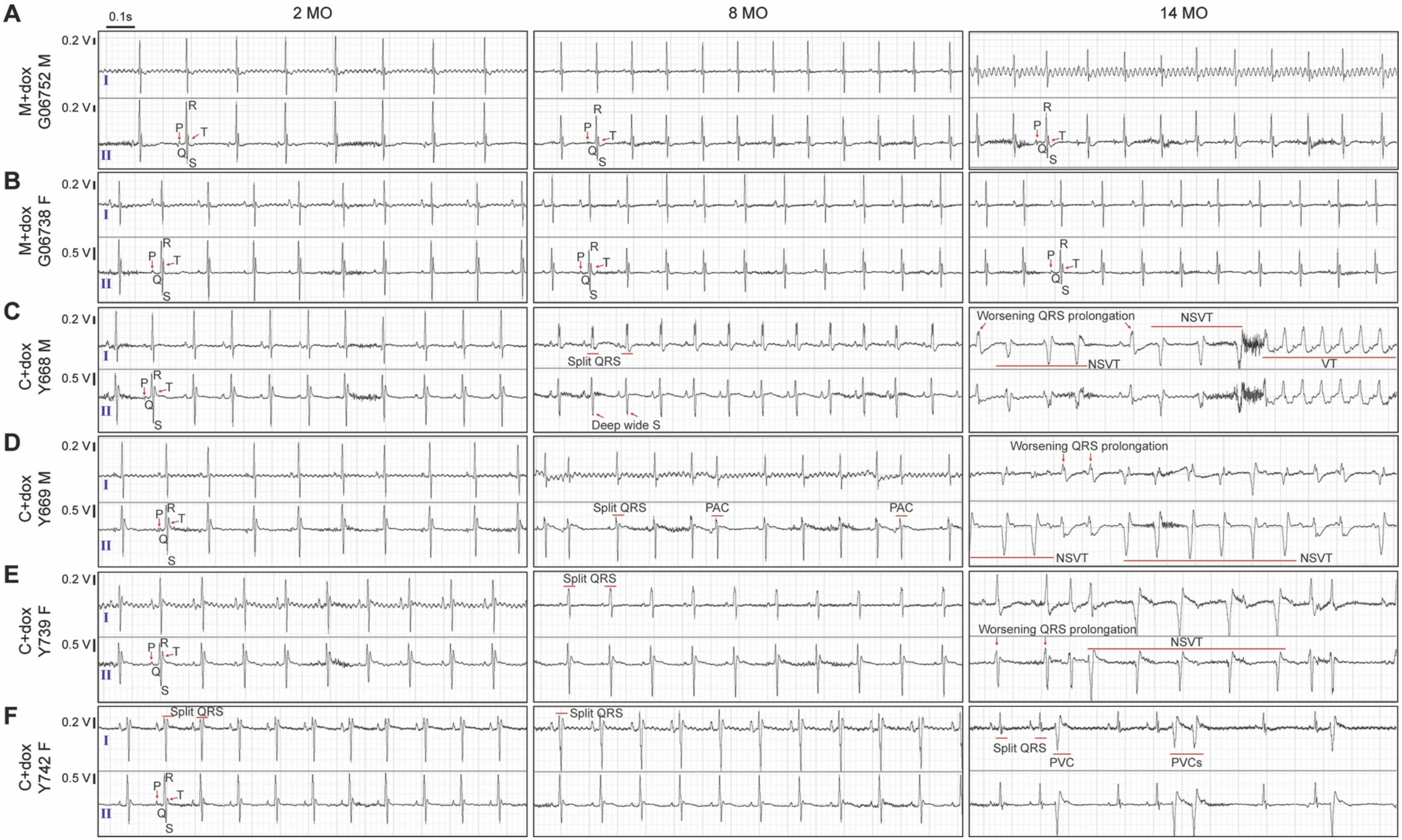

Supplemental Figure 4. Increased prevalence of abnormal cardiac rhythms in CUG960 +dox mice during long-term CUG_{exp} expression. Representative ECG tracings of MHCrtTA +dox (M+dox) animals **(A)** male G06752 and **(B)** female G06738, and CUG960 +dox (C+dox) animals **(C)** male Y668, **(D)** male Y669, **(E)** female Y739, and **(F)** female Y742, at 2, 8, and 14 months of age. Images were taken from Lead I (left arm to right arm) and II (left leg to right arm) after high-pass filter (cut off frequency: 5 Hz) were applied

to remove low-frequency artifacts and prevent base shift. PVC: premature ventricular contraction. NSVT: non-sustained ventricular tachycardia. VT: sustained ventricular tachycardia.

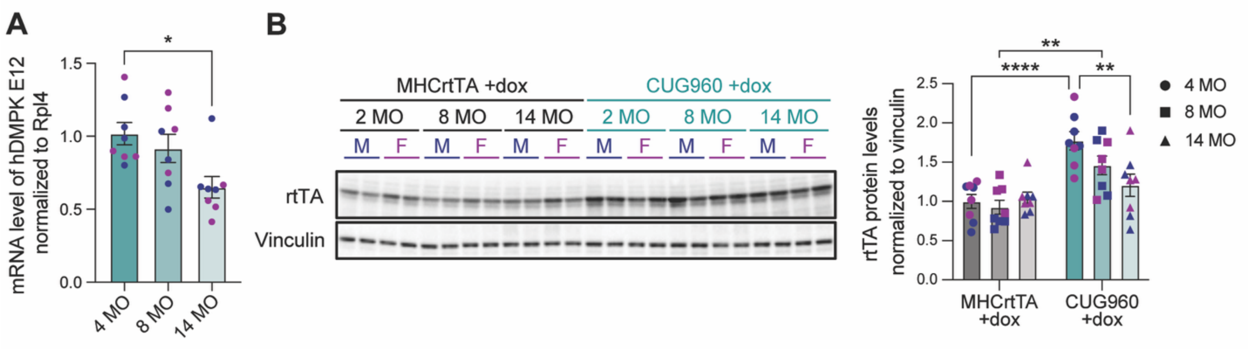

Supplemental Figure 5. CUG960 +dox mice with long-term CUG$_{exp}$ RNA expression showed decreased expression of CUG$_{exp}$ RNA and rtTA protein. (A) RT-qPCR analysis of transgene mRNA expression normalized to *Rpl4* mRNA in left ventricles of CUG960 +dox mice. **(B)** Western blot analysis of rtTA protein expression in left ventricular protein extracts. Representative western blot images of rtTA and Vinculin are shown with quantitation of protein levels shown on the right. *n*=8 animals per timepoint (4 males and 4 females). Males (blue symbols) and females (purple symbols) are indicated. Data represent the mean ± SEM and were analyzed using ordinary one-way ANOVA (for Figure A) or two -way ANOVA (for Figure B) followed by Tukey's multiple comparisons test. *p<0.05, **p<0.01, ****p<0.0001.

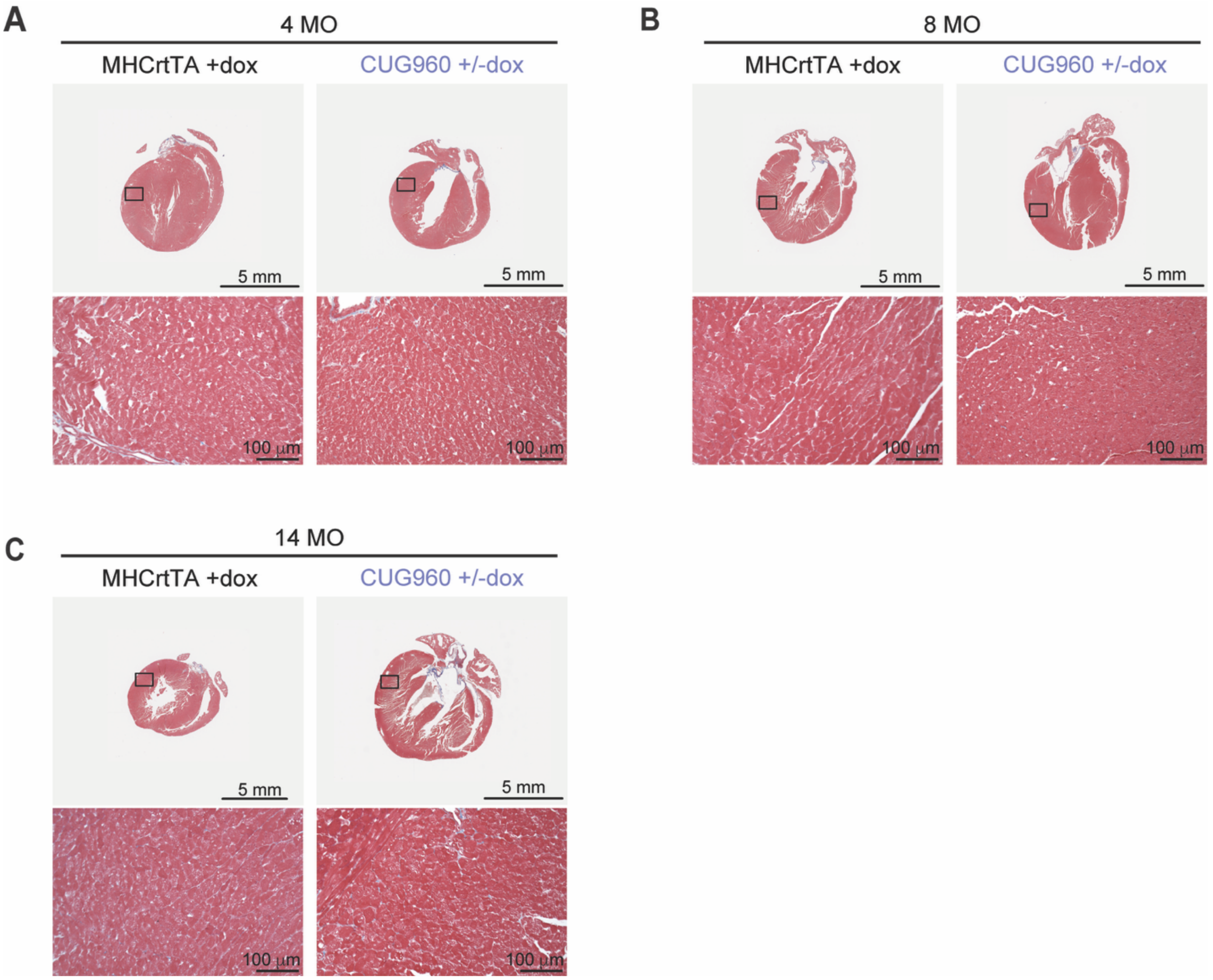

Supplemental Figure 6. Fibrosis was not detected in hearts of short- and medium-term cohorts at the 2-month off-dox timepoint. Representative images of trichrome staining from four-chamber heart sections of male MHCrtTA +dox control and CUG960 +/-dox mice at **(A)** 4 months, **(B)** 8 months, and **(C)** 14 months of age. Two animals were examined per sex per group. Section thickness: 4 microns. Scale bar for the whole-heart images is 5 mm and for the zoomed in images is 100 µm.

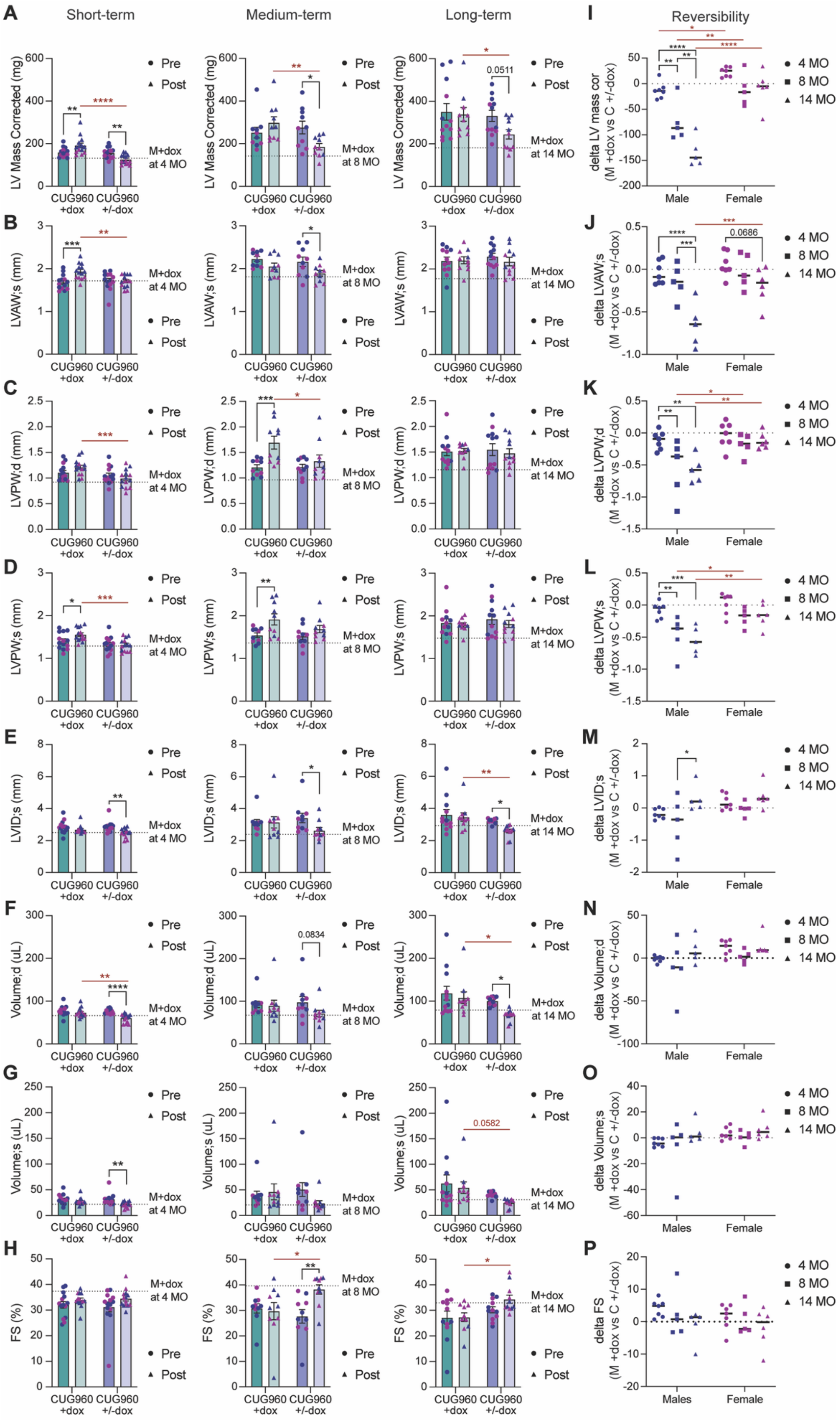

A
Short-term
Medium-term
Long-term
I
Reversibility
LV Mass Corrected (mg)
Pre
Post
M+dox at 4 MO
M+dox at 8 MO
M+dox at 14 MO
CUG960 +dox
CUG960 +/-dox
0.0511
delta LV mass cor (M +dox vs C +/-dox)
4 MO
8 MO
14 MO
Male
Female
B
LVAW;s (mm)
J
delta LVAW;s (M +dox vs C +/-dox)
0.0686
C
LVPW;d (mm)
K
delta LVPW;d (M +dox vs C +/-dox)
D
LVPW;s (mm)
L
delta LVPW;s (M +dox vs C +/-dox)
E
LVID;s (mm)
M
delta LVID;s (M +dox vs C +/-dox)
F
Volume;d (uL)
0.0834
N
delta Volume;d (M +dox vs C +/-dox)
G
Volume;s (uL)
0.0582
O
delta Volume;s (M +dox vs C +/-dox)
Males
H
FS (%)
P
delta FS (M +dox vs C +/-dox)

Supplemental Figure 7. Reduced reversibility of LV structural parameters following long-term CUG_{exp} RNA expression was observed compared to short-term cohorts. M-mode echocardiography was performed on CUG960 +dox (teal bars) and CUG960 +/-dox (purple bars) mice with short-, medium-, and long-term of CUG_{exp} RNA expression. Dark teal and dark purple bars are the results from parallel cohorts on dox for short-, medium- or long-term that either remained on dox for 2 additional months (light teal) or were taken off dox for 2 months (light purple). Dark and light teal bars are from the same animals as are dark and light purple bars. **(A)** LV mass; **(B)** LV anterior wall thickness in systole (LVAW; s); LV posterior wall thickness **(C)** in diastole (LVPW; d) and **(D)** in systole (LVPW; s); **(E)** LV internal diameter in systole (LVID; s); volume **(F)** in diastole (volume; d) and **(G)** in systole (Volume; s) and **(H)** fractional shortening (FS) were evaluated. The dotted lines indicate average of MHCrtTA+dox controls (not expressing CUG_{exp} RNA) at the age indicated. **(I-P)** Reversibility of each parameter was calculated by subtracting the values of CUG960 +/-dox from averaged MHCrtTA +dox control value of the corresponding parameter and timepoint. $n \geq 4$ per group, timepoint and sex. Data present the mean ± SEM and were analyzed using two-way ANOVA followed by Tukey's multiple comparisons test. *$p<0.05$, **$p<0.01$, ***$p<0.001$, ****$p<0.0001$.

Mice with Cardiac Electrophysiological Disorders							
Group	Sex	Short-term		Medium-term		Long-term	
		2 MO (Pre)	4 MO (Post)	6 MO (Pre)	8 MO (Post)	12 MO (Pre)	14 MO (Post)
CUG960 +dox	Male	2/9 (22.2%)	5/9 (55.6%)	5/6 (83.3%)	6/6 (100%)	4/4 (100%)	3/3 (100%)
	Female	1/5 (20%)	2/5 (40%)	3/4 (75%)	3/4 (75%)	6/8 (75%)	8/8 (100%)
CUG960 +/-dox	Male	3/7 (42.9%)	1/7 (14.3%)	5/5 (100%)	2/5 (40%)	6/6 (100%)	4/5 (80%)
	Female	5/7 (71.4%)	1/7 (14.3%)	4/5 (80%)	2/5 (40%)	5/6 (83.3%)	2/6 (33.3%)

Supplemental Figure 8. Reduced reversibility of cardiac electrophysiological disorders following long-term CUG_{exp} expression compared to short-term cohorts after dox removal. Numbers of animals per sex showing cardiac electrophysiological disorders (IVCD and/or arrhythmias) on surface ECG tracings.

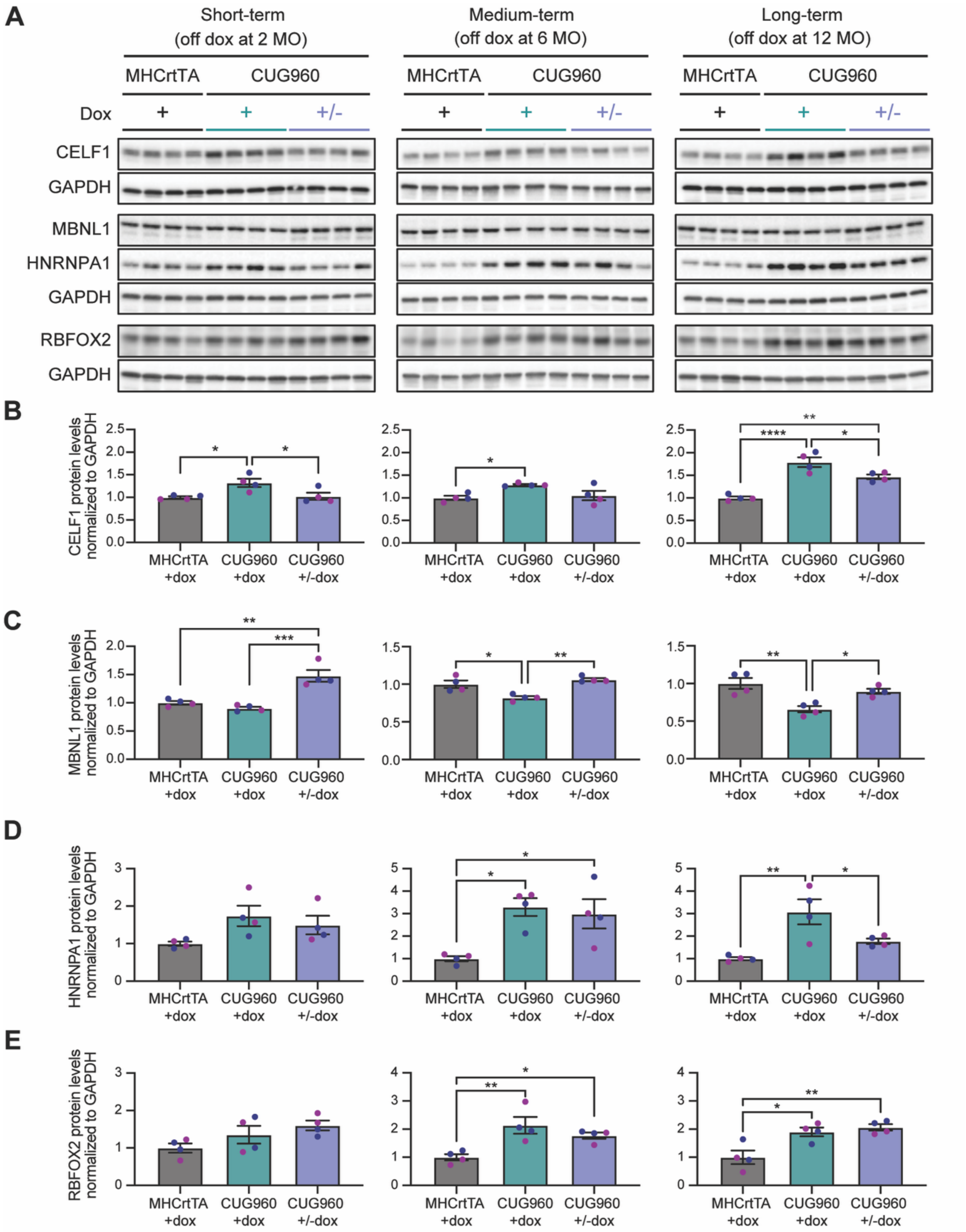

Supplemental Figure 9. Mis-regulated expression of RBPs in CUG960 +/-dox mice with long-term CUG_{exp} RNA expression showed variable levels of reversal after stopping CUG_{exp} RNA expression. (A) Ventricular protein expression was evaluated by western blotting analysis. Representative western blot images of CELF1, MBNL1, HNRNPA1, RBFOX2, and GAPDH are shown. Quantitation of **(B)** CELF1, **(C)** MBNL1, **(D)** HNRNPA1, and **(E)** RBFOX2 protein levels

normalized to GAPDH in each timepoint are shown. *n*=4 animals per cohort. Males (2, blue symbols) and females (2, purple symbols) are indicated. Data represent the means ± SEM and were analyzed using one-way ANOVA followed by Tukey's multiple comparisons test. * p<0.05, ** p<0.01, *** p<0.001, ****p<0.0001.

Supplementary Table 1. List of primers used for mouse genotyping

TREDT960I_F	TGCCGTCGAGTTTACTCCCTATCA
TREDT960I_R	AATTCTCCAGGCGATCTGACGGTT
MHCrtTA_F	CTGGGTTGCGTGTTGGAAGATC
MHCrtTA_R	GTGGGAGATCGAGCAGGCCCTCG

Supplementary Table 2. List of primers used for RT-qPCRs in mouse tissue

*hDMPK*_E15_down_F	CCGTGTTCCATCCTCCAC
*hDMPK*_E15_down_R	CCGAGTAAGCAGGCAGAGAT
*mRpl4*_F	CGCTGGTGGTTGAAGATAAGG
*mRpl4*_R	CGGTTTCTCATTTTGCCCTTG
*mPostn*_qPCR_F	CCTGTAAGAACTGGTATCAAGGT
*mPostn*_qPCR_R	CCTTTCATCCCTTCCATTCTCA
*mCol1a1*_qPCR_F	CTTCACCTACAGCACCCTTGTG
*mCol1a1*_qPCR_R	TGACTGTCTTGCCCCAAGTTC

Supplementary Table 3. List of primers used for RT-PCRs in mouse tissue

*mTnnt2*_E5_F	GTACGAGGAGGAACAGGAAG
*mTnnt2*_E5_R	CCAGCCTCCTCCTCCTCC
*mScn5a*_E6A_F	GTCGGCTCTTCGAACTTTCA
*mScn5a*_E6B_F	CGGGCCCTGAAAACTATATC
*mScn5a*_E7_R	CCAATGAGGGCAAAGACACT
*mRyr2*_E4, 5_F	CGGACCTGTCTATCTGCACCTTTGT
*mRyr2*_E4, 5_R	CATACCACTGTAGGAATGGCGTAGCA
*mCacna1s*_E29_F	GAGATCCTTGGAATGTGTTTGACTTCCT
*mCacna1s*_E29_R	GGTTCAGCAGCTTGACCAGTCTCAT
*mLdb3*_E11_F	GCAAGACCCTGATGAAGAGGCTCTG
*mLdb3*_E11_R	CAGTGGTTACGACCCGAGGCTTG
*mMyom1*_E18_F	TGAATGCAGCTGGACTTAGC
*mMyom1*_E18_R	CCATCCAAGAACCATGGAGT
