# Supplementary material for: Progressive cardiac phenotypes and reduced reversibility from long-term CUGexp RNA expression in a DM1 mouse model
Source: JCI Insight. 2026 Mar 19;11(9):e204278. doi: 10.1172/jci.insight.204278 (PMC13232024; doi:10.1172/jci.insight.204278)

**Full unedited gels for Figure 4A**

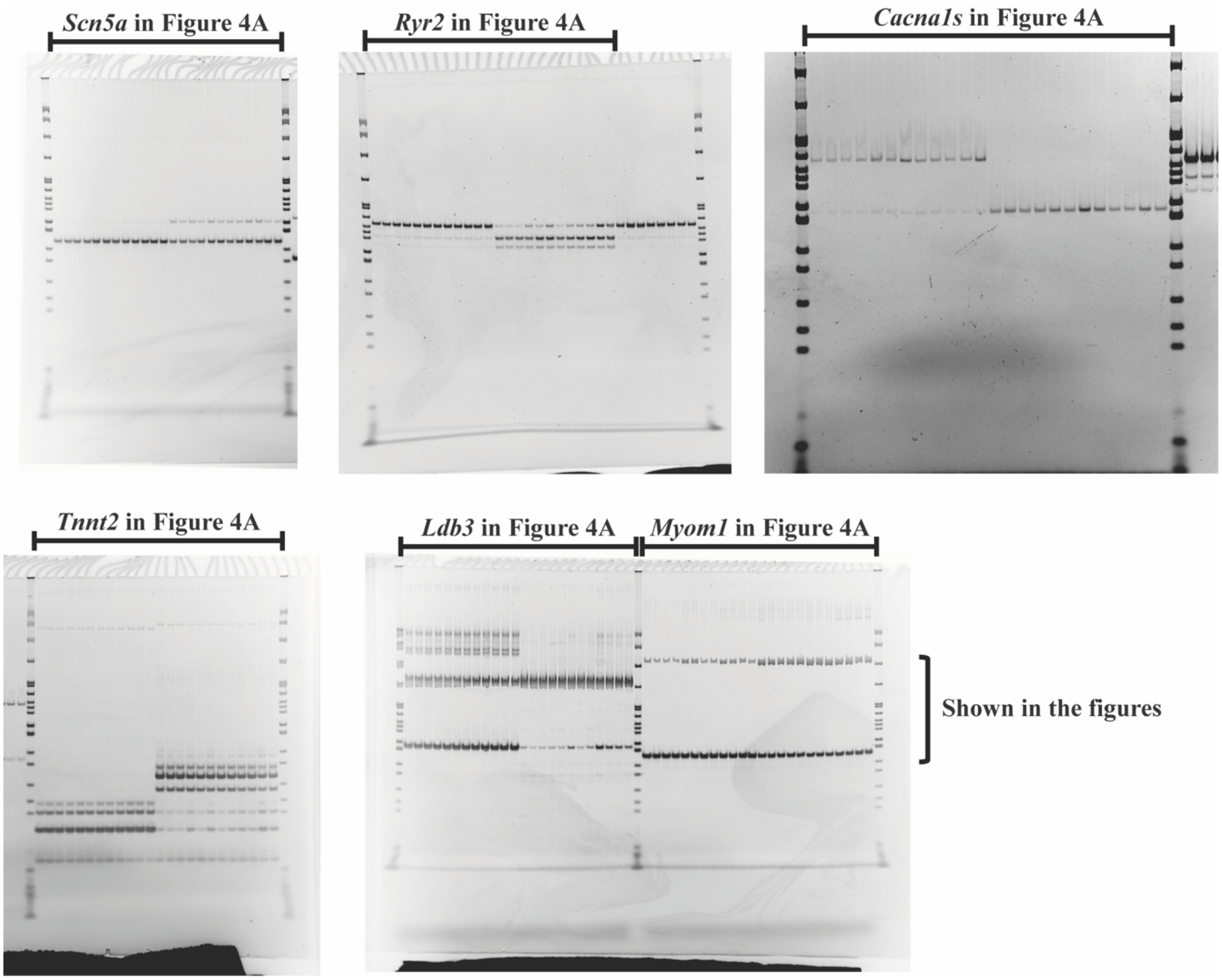

**Full unedited blots for Figure 4C (left: with marker; right: image used in figure)**

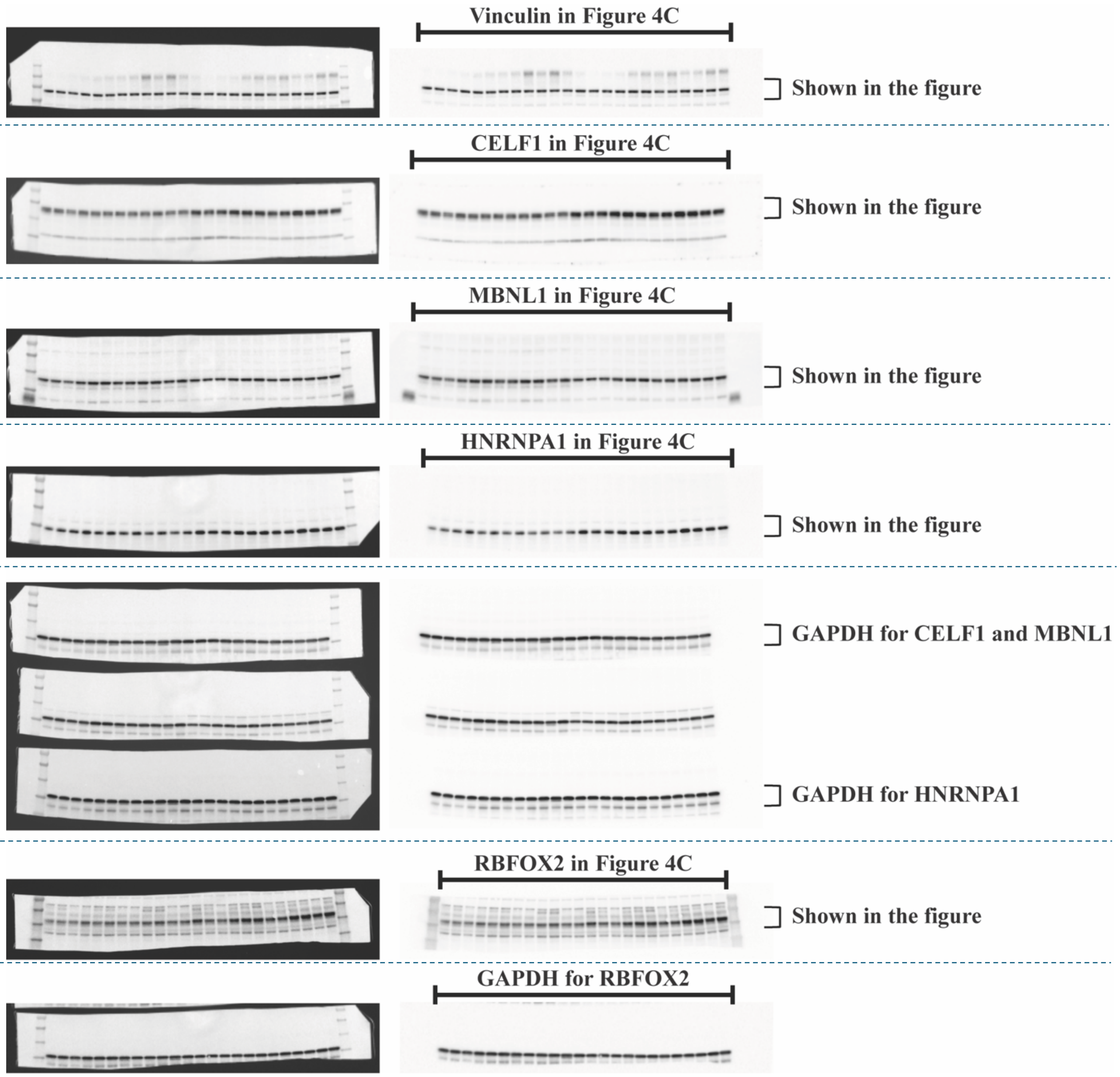

**Full unedited blots for Supplemental Figure 5B (left: with marker; right: image used in figure)**

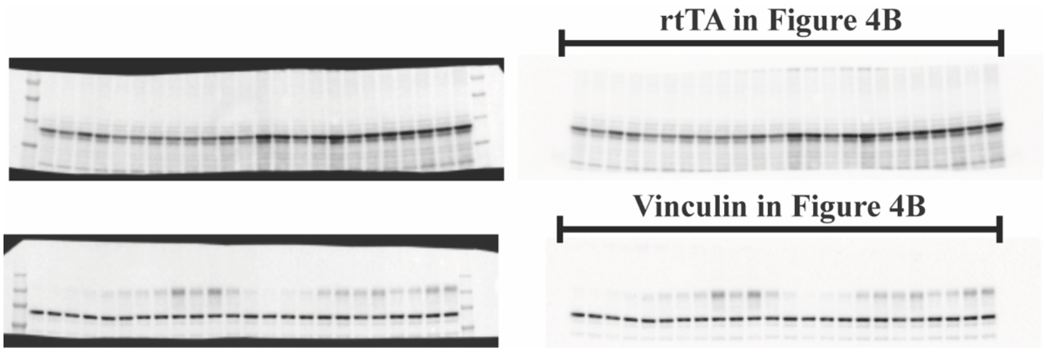

**Full unedited gels for Figure 8**

*Tnnt2* in Figure 8A

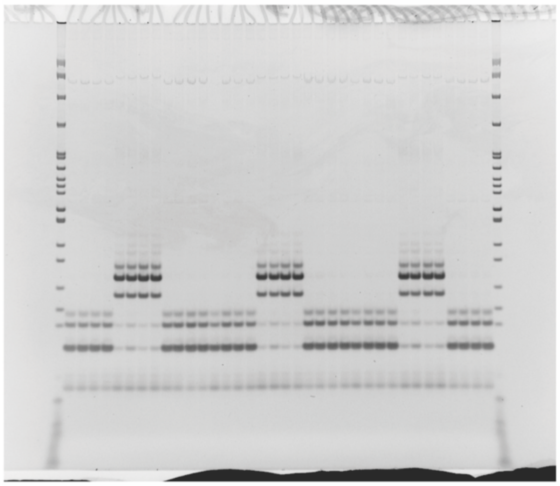

*Scn5a* in Figure 8B

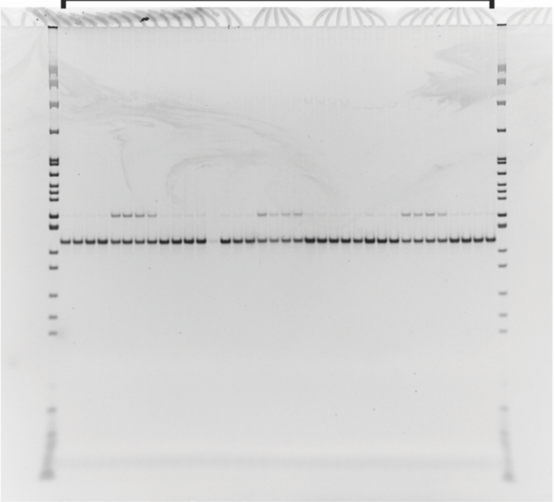

*Cacna1s* in Figure 8C

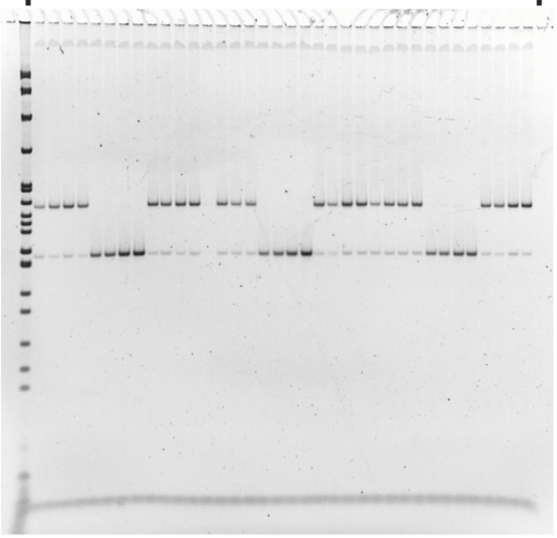

*Ryr2* in Figure 8D

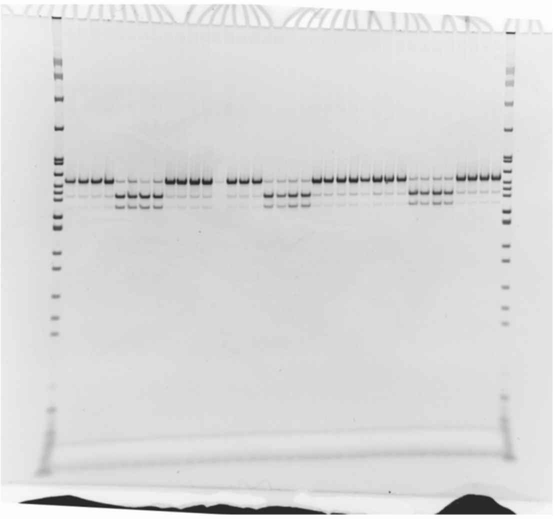

*Myom1* in Figure 8E

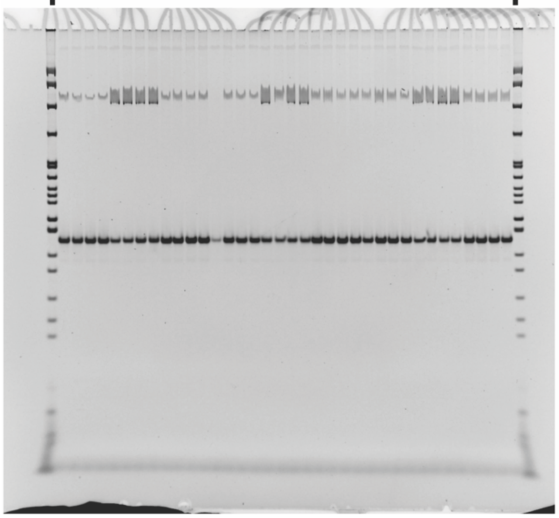

*Ldb3* in Figure 8F

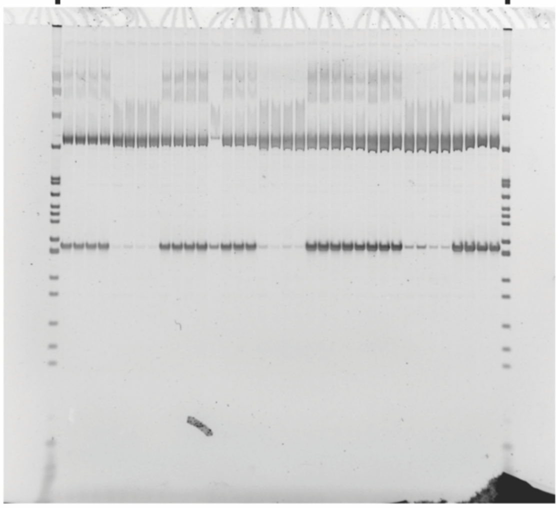

**Full unedited blots for Supplemental Figure 9A (left: with marker; right: image used in figure)**

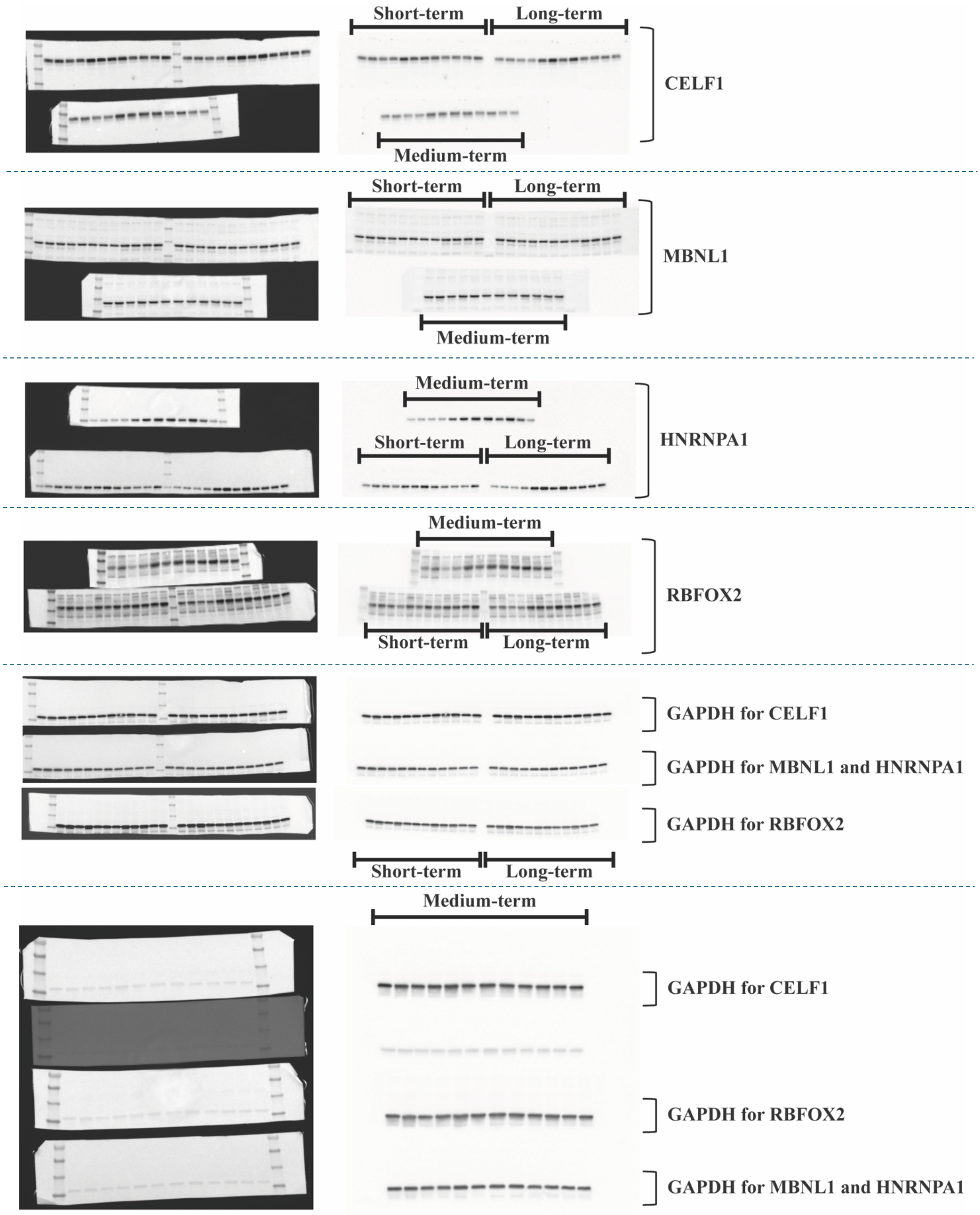

Supplement: Unedited blot and gel images [file jciinsight-11-204278-s112.pdf]
